# Diels‐Alder Click Chemistry as a Dynamic‐Covalent Crosslinking Method in Spheroid‐Encapsulating Hydrogels for Cartilage Engineering

**DOI:** 10.1002/adhm.202505013

**Published:** 2026-02-20

**Authors:** Sanne M. van de Looij, Antonia G. Vasilopoulou, Lennard Spauwen, Antoinette van den Dikkenberg, Jasmijn V. Korpershoek, Mylene de Ruijter, Jos Malda, Bas G. P. van Ravensteijn, Tina Vermonden

**Affiliations:** ^1^ Division of Pharmaceutics Utrecht Institute for Pharmaceutical Sciences (UIPS) Utrecht University Utrecht the Netherlands; ^2^ Department of Orthopedics University Medical Center Utrecht Utrecht the Netherlands; ^3^ Regenerative Medicine Center Utrecht Utrecht the Netherlands; ^4^ HU University of Applied Sciences Utrecht Utrecht the Netherlands; ^5^ Department of Orthopedic Surgery Mayo Clinic Rochester Rochester Minnesota USA; ^6^ Department of Clinical Sciences Faculty of Veterinary Medicine Utrecht University Utrecht the Netherlands

**Keywords:** cartilage engineering, cell‐encapsulation, Diels‐Alder click‐chemistry, hydrogels, multicellular cartilage spheroids

## Abstract

In cartilage tissue engineering, there is a growing interest in dynamic hydrogels that promote spheroid fusion and cartilaginous matrix deposition, while maintaining sufficient stability for long‐term construct maturation. In this study, Diels‐Alder click chemistry is employed as a dynamic‐covalent crosslinking method to create hydrogels composed of hyaluronic acid, gelatin, and PEG. By adjusting the pH during crosslinking, the tuneability of hydrogel stiffness and stability at pH values around the physiological pH of native cartilage is demonstrated. This pH modulation does not compromise hydrogel functionality, as encapsulated equine articular cartilage progenitor cell spheroids remain viable and functional for a culture period of 28 days. The hydrogel environment supports the deposition of cartilaginous extracellular matrix components, including collagens and sulphated glycosaminoglycans. Enhanced chondrogenesis and deposition of collagen type II are observed at higher spheroid concentrations, corresponding to inter‐spheroid distances of 100–150 µm following hydrogel swelling, compared to lower concentrations at a distance of >500 µm. To further improve construct robustness, the hydrogel constructs can be reinforced on day 1 with a melt electrowritten scaffold, increasing the compressive modulus 100‐fold by day 28 compared to non‐reinforced constructs, highlighting the potential of this system for engineering cartilage implants.

## Graphical abstract

This research shows the development of hydrogels with Diels‐Alder click chemistry for engineering cartilage‐like tissue. The hydrogels support cartilage spheroids which could be cultured for at least 28 days. Furthermore, the spheroids showed a tendency to fuse together into a more consistent construct, and produced important components needed for functional cartilage generation.

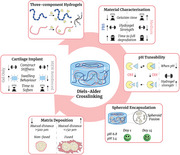

## 1 Introduction

Articular cartilage is a connective tissue that covers the end of articulating bones, where it provides a near frictionless movement [1, 2, 3]. Slow‐dividing chondrocytes maintain the highly hydrated extracellular matrix (ECM) of the tissue, a firm tissue containing primarily water, structural collagens, and water‐attracting proteoglycans. Due to the avascular and alymphatic nature of articular cartilage, chondrocytes rely exclusively on the long‐range diffusion of required nutrients and waste products through the ECM. Partially because of this reduced access to nutrients, cartilage has a limited capacity for self‐regeneration. In healthy individuals, this poses no problem, as the tissue is preserved by a carefully regulated homeostasis within the joints [3, 4]. However, the precarious balance between anabolic processes and catabolic activities of proteolytic enzymes is lost in the case of highly prevalent rheumatic and degenerative diseases, leading to slow cartilage deterioration and chronic pain [1, 3].

The use of hydrogels as scaffolds for cartilage tissue engineering has been extensively studied [5, 6, 7, 8, 9]. Generally, cells with chondrogenic potential are embedded within a temporary 3D polymer network, where they are retained in a gel‐like structure. The hydrogel provides a local supportive environment to the cells to aid in the deposition of ECM over time, causing a matrix turnover in which cell‐made ECM replaces the polymer network as the scaffold degrades. In order to induce this process, the hydrogel scaffold should be fully biodegradable in a period of one to several months, cause no cytotoxicity, and have suitable mechanical properties [4, 10]. Suitable can herein be defined as having a stiffness in the range of healthy human pericellular matrix, with a compressive modulus between 27 and 205 kPa [11]. A gel that is too stiff would not provide the appropriate mechanical stimulation, which can lead to stress shielding and induce a fibroblastic appearance in encapsulated cells [12]. Too soft gels, on the other hand, can induce unwanted adipogenic differentiation in some chondrogenic cell types, such as mesenchymal stromal cells (MSCs) [13, 14, 15]. Various aspects of the hydrogel formulation can affect these requirements, namely the type and exact composition of polymers used; the type of crosslinking chemistry employed; and the density of these crosslinks [6, 16].

There are many types of polymers that have been investigated in the context of tissue engineering and regenerative medicine. Herein there is the distinction between natural and synthetic polymers, as well as chemically modified natural polymers. Hyaluronic acid, an example of a natural polymer, is a non‐sulphated glycosaminoglycan found within cartilage ECM [6, 17, 18]. Likely because of CD44‐mediated cellular interaction [19], naturally occurring HA has been found to play an important role in regulating cellular receptor binding and cell migration and could also increase chondrogenesis [18]. However, many studies highlight the limitations of unmodified HA as the sole component of a 3D construct due to unfavorable bulk mechanical properties and fast degradation in vivo. Therefore, efforts have been made to chemically improve the mechanical characteristics of HA‐containing hydrogels, while still harnessing the desirable cell affinity [17].

Although HA in theory has promising cell adhesion properties, it was found that hydrogels containing only HA were not suitable for seeding and growing cells in a monolayer on the hydrogel surface without adding a cell‐adhesive coating [18]. While these findings for 2D adhesion are not directly translatable to encapsulation strategies, they are useful keys to achieve the goal of a biological supportive environment where the cells are in a native‐reflecting 3D shape and are maintained in the supporting system while producing their matrix. Therefore, to improve the biocompatibility of hydrogel formulations and increase the amount of adhesive domains, gelatin is often incorporated into the polymer network [17, 18, 20, 21].

The crosslinking chemistry used to form the polymer network also greatly affects hydrogel properties. A wide variety of crosslinking methods have been investigated in the context of tissue engineering, ranging from highly static chemical linkages to extremely dynamic physical interactions [22]. ECM deposition by encapsulated cartilage spheroids was found to be promoted within a more viscous environment compared to a highly elastic environment [23], indicating that the use of a polymer crosslinking chemistry that provides a certain degree of reconfigurability is therefore desired. However, hydrogels crosslinked by fully dynamic chemistries such as host–guest interaction or boronic esters are often unstable [24, 25]. As it is important that formed hydrogels are shape‐retaining and conform to the requirement of suitable mechanical stiffness, a dynamic‐covalent crosslinking chemistry could be applied for this purpose. The Diels‐Alder (DA) click‐reaction is a reversible [4+2] cycloaddition between a diene and a dienophile, and for biological applications most often a furan and maleimide are used [22]. DA is an attractive choice for the preparation of cytocompatible hydrogel systems due to its reversibility and the fact that the reaction occurs under physiological conditions without the need for potentially toxic catalysts or initiators [22, 26]. Yet, despite these attractive qualities, research has been primarily focused on applying DA‐crosslinked hydrogels as drug delivery depots [26, 27, 28, 29, 30] as opposed to cell encapsulation scaffolds [22, 31, 32]. The limited research in the context of encapsulating cells in DA hydrogels for the purpose of tissue engineering could be due to practical limitations such as the typically rather long gelation time [31, 33]; the relatively fast degradation times in cell culture medium [34, 35]; as well as the side reactions maleimides can undergo with thiol‐containing biomolecules such as cysteines in Michael‐type 1,4‐addition reactions [22, 34]. Nimmo et al. developed a DA‐based hydrogel using hyaluronic acid‐furan and PEG‐bis‐maleimide, achieving a maximum stiffness (*G*’) of 679 Pa [36]. This stiffness was deemed sufficient for engineering of brain and nerve tissue, but is considerably soft in the context of cartilage tissue engineering. Yu et al. developed a similar hydrogel for the purpose of cartilage tissue engineering [31], and reported gelation times of more than 50 min at physiological temperature, and a swelling factor of over 12.5. More recently, Fatima and Almeida added collagen‐furan to the formulation, and developed hydrogels with tunable stiffnesses [37]. However, gelation times of 3–6 h and times to full gelation of around 20 h were reported, vastly limiting the use in tissue engineering. While more hydrogels that have DA as a crosslink method are reported in literature, they often use another crosslink method alongside DA to improve gelation time, stiffness or degradability [35, 38, 39, 40]. However, outside of tissue engineering applications there are examples where a gelation time of as low as five minutes was reported when forming HA/PEG based DA hydrogels in phosphate‐buffered saline (PBS), with a corresponding stiffness of more than 60 kPa^26^. This highlights the potential of the DA reaction in hydrogels, and the need for the optimization of this crosslinking method under culturing conditions for the purpose of cell‐encapsulating hydrogels in tissue engineering.

With a stiffness on the lower end of the stiffness range of the articular cartilage pericellular matrix (0.02–0.2 MPa), hydrogels could provide the appropriate conditions for growth and differentiation in the local environment of encapsulated cells and multicellular spheroids. However, although the results regarding the compressive stiffness of native human cartilage ECM vary to a great extent, with measured values ranging from 0.1 to 1.6 MPa [11, 41], the overall consensus is that the cartilage ECM is at least an order of magnitude stiffer than most hydrogels. Consequently, the bulk mechanical stiffness of hydrogels is generally too soft to withstand the physiological load that articular cartilage is subjected to in the body. By incorporating a melt electrowritten (MEW) polycaprolactone (PCL) scaffold, soft hydrogels can be reinforced to enhance the mechanical properties [42, 43], thereby enhancing their applicability as a cartilage implant. MEW scaffolds contain individual fibers with a diameter of typically 10 µm, resulting in an efficient reinforcing strategy (7% of polymer fiber resulted in a 54‐fold increase in mechanical stiffness) without stress shielding [44].

This study aims to investigate the potential of Diels‐Alder hydrogels to facilitate cartilage progenitor spheroid cell encapsulation, differentiation, and fusion towards cartilage tissue formation. To this end, we first investigate the material properties of various three‐component hydrogel formulations consisting of furan‐modified hyaluronic acid (HAFU), gelatin‐furan (GelFU), and 4‐arm PEG‐maleimide (4APM), when formed in either PBS or cell culture medium. Herein, special attention is paid to gelation kinetics, mechanical strength, and hydrogel degradation. Next, we test whether pH alterations of typical culturing medium could improve these aforementioned material properties of formed hydrogels. Furthermore, the potential prospect of using HAFU/GelFU/4APM hydrogels for the use in cartilage engineering is examined by encapsulating articular cartilage progenitor cell (ACPC) spheroids and culturing these constructs for 28 days to show prolonged cytocompatibility and matrix deposition. In particular, we highlight the importance of a small inter‐spheroid distance during culturing to enhance spheroid fusion to obtain a cartilage‐like tissue as the Diels‐Alder hydrogel softens and degrades. Lastly, we reinforce the DA hydrogel with a MEW PCL scaffold for translation of the construct towards a cartilage implant, showing strong bulk mechanical properties with a soft and dynamic environment for encapsulated cells. In literature reported until now, hydrogels crosslinked solely with Diels‐Alder chemistry have strong limitations for cartilage tissue engineering in terms of mechanical properties. The novelty of this research lies in the optimization of the DA hydrogel formation under cell culture‐relevant conditions by tuning the total polymer content and crosslinking pH. By tweaking the crosslinking conditions, we form a cell‐friendly hydrogel with a gelation time and bulk stiffness suitable for cartilage tissue engineering, and long enough degradation times to allow for construct maturation into a cartilage‐like tissue.

## 2 Results and Discussion

### 2.1 Polymer Modification and Hydrogel Design

**FIGURE 1:**
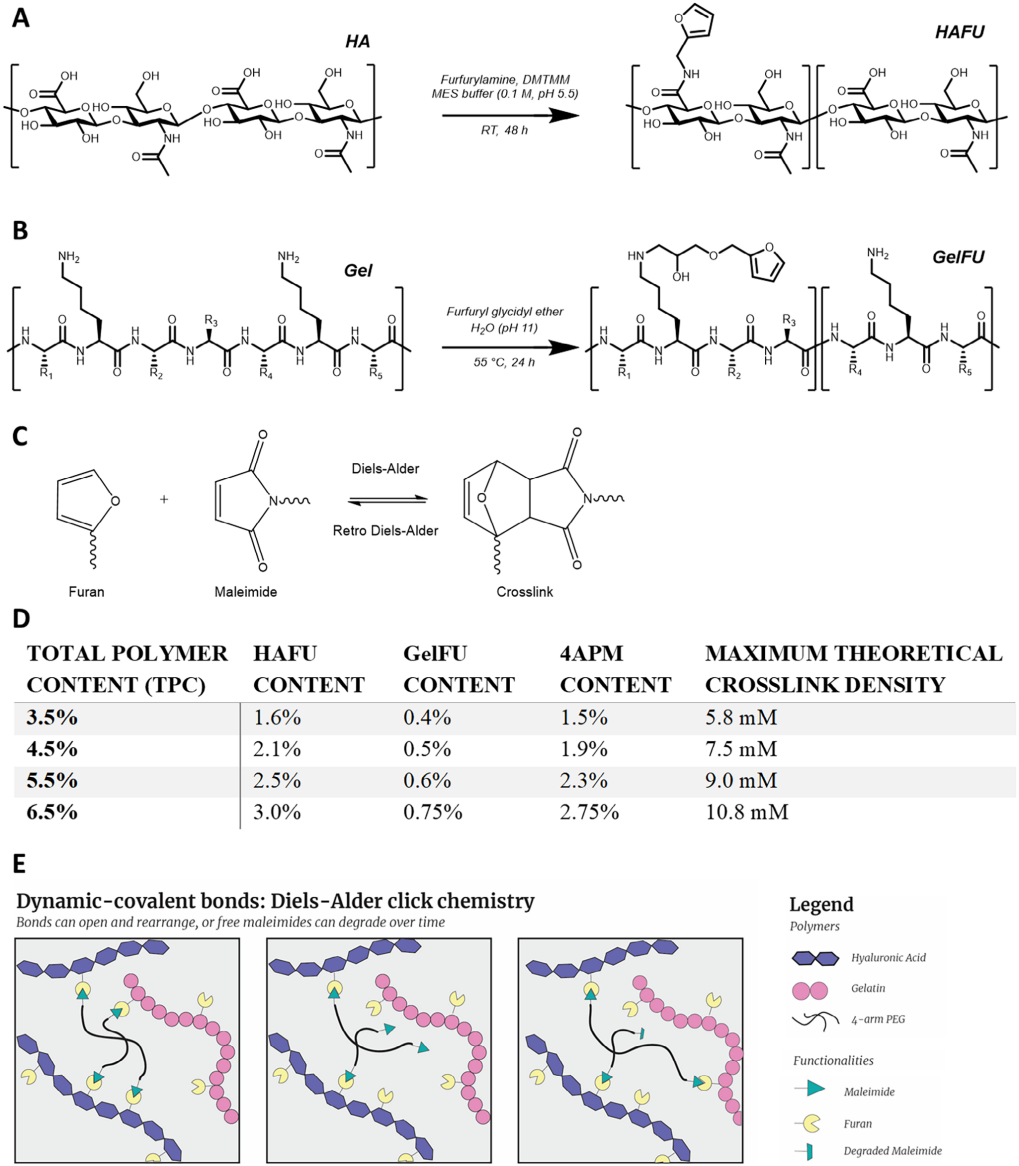
(A) Chemical structures of the one‐step synthesis of HA and furfurylamine to obtain HAFU. (B) Chemical structures of the one‐step synthesis of gelatin and furfuryl glycidyl ether to obtain GelFU. (C) Chemical structures of furan, maleimide and the Diels‐Alder adduct. (D) Compositions of different hydrogel formulations tested. (E) Schematic overview of dynamicity of Diels‐Alder crosslinks in hydrogels. Over time, crosslinks can open and rearrange, or free maleimides can degrade, leading to a dynamic environment.

HA with a molecular weight of 381–391 kDa was chosen over lower molecular weight HA due to the reported disadvantages of low molecular weight HA in terms of immunogenicity and inflammatory responses [45, 46], whereas higher molecular weight HA was not chosen because of its high viscosity and thereby decreasing workability. Furan‐derivatized HA was prepared following a previously optimized method by Ilochonwu et al. [26], utilizing a coupling agent to activate the carboxyl groups of HA, followed by a coupling reaction to the primary amine of furfurylamine (Figure 1A). ^1^H‐NMR analysis demonstrated successful grafting of furan on HA with a degree of functionalization (DF) of 65% (Figure S1), in which the DF is defined as the number of furan residues per 100 HA disaccharide units. Gelatin was modified to contain furan groups by reacting furfuryl glycidyl ether with primary amines on lysine residues (Figure 1B). The reaction was executed in water at a pH of 11 to ensure high reactivity of the unprotonated amines on lysine side chains (p*K*a 10.54). A 10‐fold excess of furfuryl glycidyl ether compared to available lysine residues was added to address the partial loss of the epoxide due to base‐catalyzed epoxide ring opening at this pH. As shown in Figure S2, ^1^H NMR analysis confirmed the presence of furan groups after purification, whereas a colorimetric assay found 91.3% of lysine residues were functionalized, leading to a modification of 0.22 mmol furan g^−1^ synthesized GelFU.

Using the modified polymers and commercially available 4APM, four different hydrogel compositions were prepared with increasing total polymer content (TPC, see Figure 1C,D). Herein, two factors were always kept constant, namely the mass ratio of HAFU to GelFU (4:1); and the molar ratio of furan to maleimide (5:1). Gelatin was added to the formulation to incorporate cell‐adhesive properties, though the final gelatin content was minimized for translational purposes, with only a presence of 0.4 to 0.75% in the hydrogel formulation (Figure 1D). The molar ratio of furan to maleimide was selected based on the optimization done in previous work by Ilochonwu et al., who found faster gelation times and slower degradation kinetics than at a molar ratio of 1:1 due to the ample availability of furan groups for each maleimide moiety, important in the dynamic nature of the crosslinks (Figure 1E) [26]. The degradability of the DA hydrogels is likely a direct consequence of the dynamicity of the DA reaction [47]. Although under physiological conditions, the dynamic equilibrium of the DA reaction lies primarily on the side of the cycloaddition product, there will always be a small amount of free furan and maleimide groups present. If present in close proximity of each other, they can undergo the DA reaction and reform a crosslink. However, maleimides can also be hydrolyzed to unreactive maleamic acid, causing the amount of DA crosslinks to continuously decrease until full degradation and dissolution of the hydrogel [47].

### 2.2 Optimization of Mechanical Properties

**FIGURE 2:**
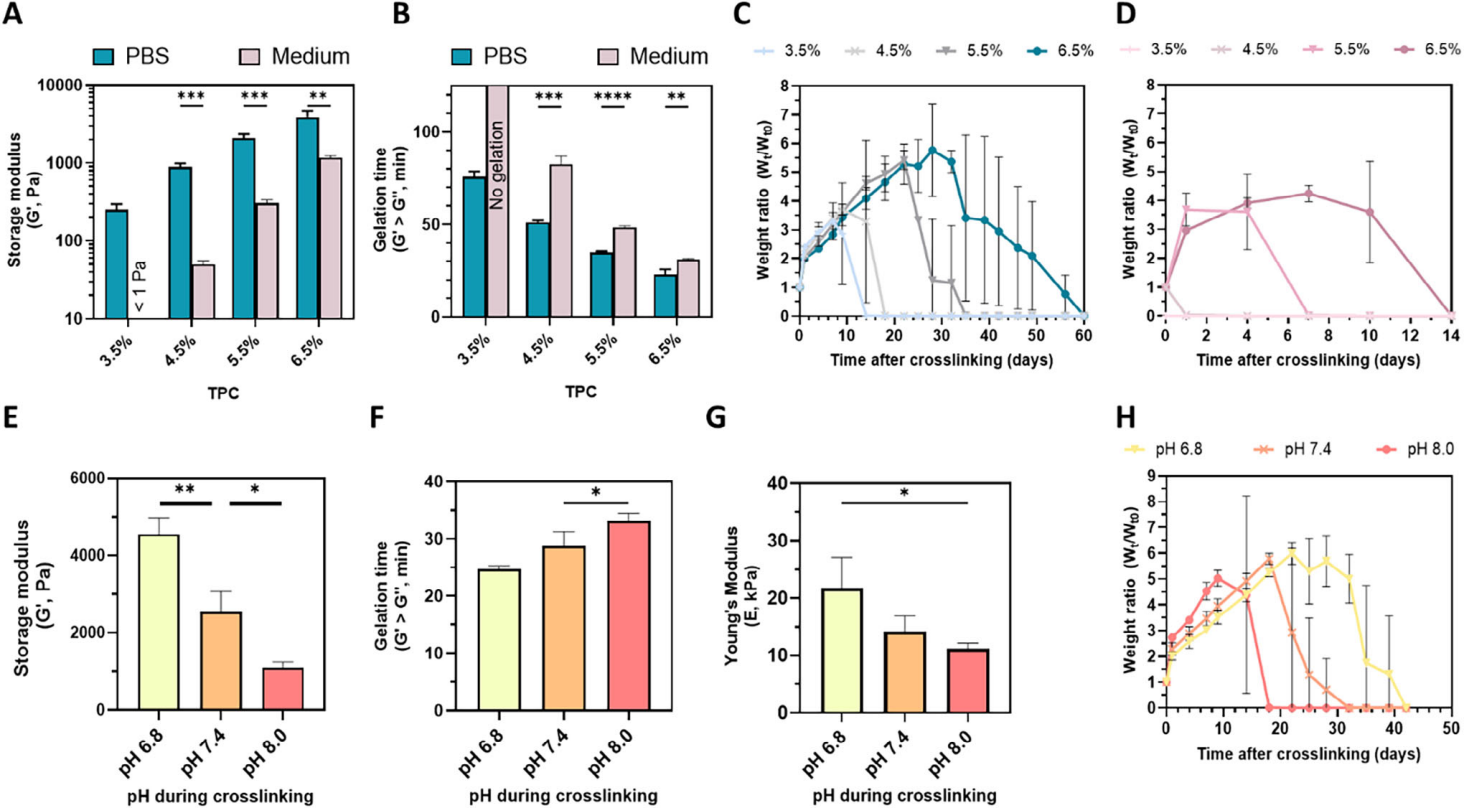
Mechanical characterization of DA hydrogels. (A) Storage moduli (*G*’, Pa) and (B) gelation times of various hydrogel formulations dissolved and formed in PBS or cell culture medium, with increasing TPC from 3.5% to 6.5%. (C) Swelling and degradation profiles of different hydrogel formulations with increasing TPC crosslinked in PBS or (D) relevant culture medium imitation. Swelling was monitored in medium for all conditions. (E) Storage moduli (*G*’, Pa), (F) Gelation times, (*G*) Young's compressive moduli (*E*, kPa), and (H) swelling and degradation profiles of 6.5% wt/wt TPC hydrogels crosslinked in medium at various pH values. Where applicable, altered pH values occur during crosslinking only (first 4 h, afterwards all samples were maintained in relevant culture medium imitation). In all graphs, values represent the average ± st.dev. of *n* = 3 or more replicates. Statistical significance in bar graphs is denoted as * (*p* < 0.05), ** (*p* < 0.01), *** (*p* < 0.001), or **** (*p* < 0.0001).

Rheological measurements were performed in the linear viscoelastic region (Figure S3) to investigate the gelation kinetics of different hydrogel compositions. The gelation time at 37°C, defined here as the time it takes for the storage modulus (*G*′) to overtake the loss modulus (*G*″) in an oscillation experiment, was shown to decrease upon increasing the TPC (Figure 2B). Similarly, it was shown that at a higher TPC, the fully formed hydrogel displayed increased stiffness (Figure 2A). Herein, the final hydrogel stiffness was defined as the maximum *G*′ after 4 h of crosslinking at 37°C. Although the *G*′ had not always plateaued after 4 h, we decided to always transfer gels from the mold to medium after 4 h crosslinking to minimize evaporation and consider the application for cell encapsulation. When encapsulating cells, the prolonged exposure of the hydrogel to air can lead to evaporation of the liquid inside the hydrogel, causing a temporarily increased TPC and potentially hampering cell viability and proliferation.

Interestingly, when comparing the gelation time and final stiffness of hydrogel formulations equal in TPC, but differing in the dissolution medium (polymers were either dissolved in PBS pH 7.4 or in relevant cell culture medium imitation (see experimental Section 4.2.3. for formulation)), we noticed a significant difference. Hydrogel formulations in which the polymers were dissolved and crosslinked in PBS, displayed significantly faster gelation and higher stiffness than the corresponding conditions in cell culture medium. This difference was especially pronounced in formulations with low TPCs, indicating less efficient crosslinking. When comparing the swelling and degradation profile of these different hydrogel formulations, the difference between PBS‐ or medium‐crosslinked conditions is again noticeable (Figure 2C,D). Even though both PBS‐ and medium crosslinked formulations were maintained in 1 mL relevant culture medium imitation after crosslinking, formulations crosslinked in PBS showed a stronger swelling capacity and over a factor of four longer degradation times than corresponding conditions crosslinked in medium. Here, it is important to note that the large standard deviations are a result of the swelling behavior. It was often noticed that full hydrogel degradation (showing a weight ratio close to 0) was preceded by extreme swelling (weight ratios close to 6 or more) in a short timeframe. Variability between samples at these specific time points then led to having highly swollen gels at the same time as fully degraded gels, thus displaying a large standard deviation in the weight ratio.

The dissimilarity in hydrogel formulations crosslinked in medium and PBS can be explained by two factors: unwanted side‐reactions or premature degradation of hydrogel components. Although the Diels‐Alder reaction is claimed to be bio‐orthogonal, Michael‐addition reactions of maleimides with thiols have often been reported in literature [48]. Although cysteines are present in most cell culturing media, the base of chondrogenic differentiation medium does not contain L‐cysteine in its native configuration. Instead, DMEM high glucose only includes the cysteine dimer L‐cystine in its formulation. To investigate whether the stable cysteine dimer could undergo Michael‐thiol reactions and cause the inferior material characteristics of medium‐crosslinked gels, L‐cystine was added to PBS in concentrations comparable to those found in DMEM (0.2 mm). The stiffness of the hydrogel at 5.5% TPC did not decrease significantly, indicating that L‐cystine does not interfere with the crosslinking reaction (Figure S4). Another potential reason for weaker gel formation in medium is related to the premature degradation of crosslinking groups. Kirchhof et al. showed how a basic environment at physiological temperatures could facilitate fast degradation of maleimides to unreactive maleamic acid during Diels‐Alder‐mediated hydrogel formation, as visualized in Figure S5 [16]. As the pH value of sodium bicarbonate‐containing medium like DMEM or αMEM rises as the medium ages and can quickly increase outside of a CO_2_‐rich environment, we adapted cell culturing medium to have a fixed pH during the polymer dissolution and crosslinking process, irrespective of the presence of CO_2_ or medium age. Instead of sodium bicarbonate, the medium was buffered with a combination of HEPES and PIPES buffer, each at a 20 mm concentration, before adapting the pH to obtain hydrogel precursor solutions with a pH of 6.8 or 7.4. With these modifications, the salt molarity in the medium was kept similar (44 mm sodium bicarbonate was replaced with 40 mm buffer salts) to regular DMEM containing sodium bicarbonate. It was shown that hydrogel crosslinking at a controlled pH value slightly lower than physiological conditions (buffered at pH 6.8 as opposed to buffered at pH 7.4), vastly improved the mechanical properties, as indicated by a 78% increase of the maximum *G*’ (Figure 2E); slight decrease of the gelation time (Figure 2F); a 50% increase of the Young's modulus (Figure 2G); and 10 d increase in time to full degradation (Figure 2H). This increased mechanical stability can thus be explained by a higher number of functional maleimide groups present to undergo the Diels‐Alder reaction, due to slower consumption of maleimides by undesired hydrolysis at lower pH. When comparing these results to pH‐controlled medium at a pH of 8.0, similar to the pH of ‘aged’ NaHCO_3_‐containing DMEM, the differences are even more striking. Altering the pH during the 4 h hydrogel formation has an effect on hydrogel crosslinking, even in the short timeframe of the hydrogel gelation time. Although no significant difference in gelation time is found when lowering the pH from pH 7.4 to pH 6.8, a clear trend is visible when including the crosslinking speed at pH 8.0. At a lower pH, the hydrogels form faster (Figure 2F). Given that the target stiffness values align with those of the native articular cartilage pericellular matrix (20‐200 kPa), the 6.5% TPC DA hydrogel crosslinked at pH 6.8 best meets this criterion, exhibiting a Young's modulus at the lower end of the desired range (21.8 ± 5.3 kPa). Regarding hydrogel stability, previous studies have shown that rapidly degrading hydrogels with a degradation time of two to three weeks in vitro promote fusion of encapsulated spheroids compared to more permanent hydrogels with static crosslinks [49]. With a time to full degradation of four to five weeks, the 6.5% TPC formulation crosslinked at pH 6.8 is slightly more stable, but falls in the targeted degradation time of one to two months.

### 2.3 ACPC Spheroids Cell Density, Size, and Viability in a Microwell System

Although hydrogels crosslinked in medium at pH 6.8 show improved hydrogel stiffness and degradation kinetics, for the purpose of tissue engineering it is important that relevant cell types are resilient against incubation at lower pH values for the 4 h period required for crosslinking. When cultured as single cells in 2D, equine articular cartilage progenitor cells (ACPCs) show no metabolism inhibition when incubated for 4 h at pH 6.0 to pH 8.0, whereas pH values lower than pH 6.0 drastically reduce cell viability (Figure S6). This is in accordance with literature, which states that in healthy cartilage the pH of the matrix surrounding chondrocytes lies between 6.6 and 6.9 [50, 51]_._

ACPCs have emerged as a promising cell source for cartilage engineering due to their stem‐cell like properties, including colony‐forming ability, extensive proliferation capacity, and strong chondrogenic differentiation potential [52]. Unlike extensively used MSCs, ACPCs do not undergo hypertrophic differentiation, an indicator for endochondral ossification, thereby overcoming a major challenge in cartilage tissue engineering [52]. It was recently demonstrated that incorporating ACPCs in cell‐loaded hydrogels, such as gelatin methacryloyl (GelMA), results in higher production of glycosaminoglycans (GAGs) and type II collagen (imperative cartilage‐matrix components) compared to chondrocytes, while the levels of type X collagen (indicator of hypertrophic differentiation) were very low [53]. Although the viability and metabolic activity of 2D cultured, single ACPCs are not affected by the pH conditions necessary for hydrogel crosslinking, exploring their behavior in a more physiologically relevant 3D environment is important for cartilage engineering strategies. Multicellular spheroids and microtissues have gained a lot of attention in cartilage engineering in recent years due to their ability to mimic the native 3D environment and promote cell–cell interactions, exhibiting improved biological features [54]. Due to their 3D architecture, spheroids display higher viability and proliferation rates, as well as better intercellular communication that boosts ECM production, as compared to standard monolayer cultures and single cell encapsulations [23, 55, 56]. Their capacity to fuse together into larger tissues can be leveraged in tissue engineering applications. To harness this potential, hydrogels play an important role in facilitating spheroid fusion while maintaining structural integrity and providing a supporting microenvironment.

**FIGURE 3:**
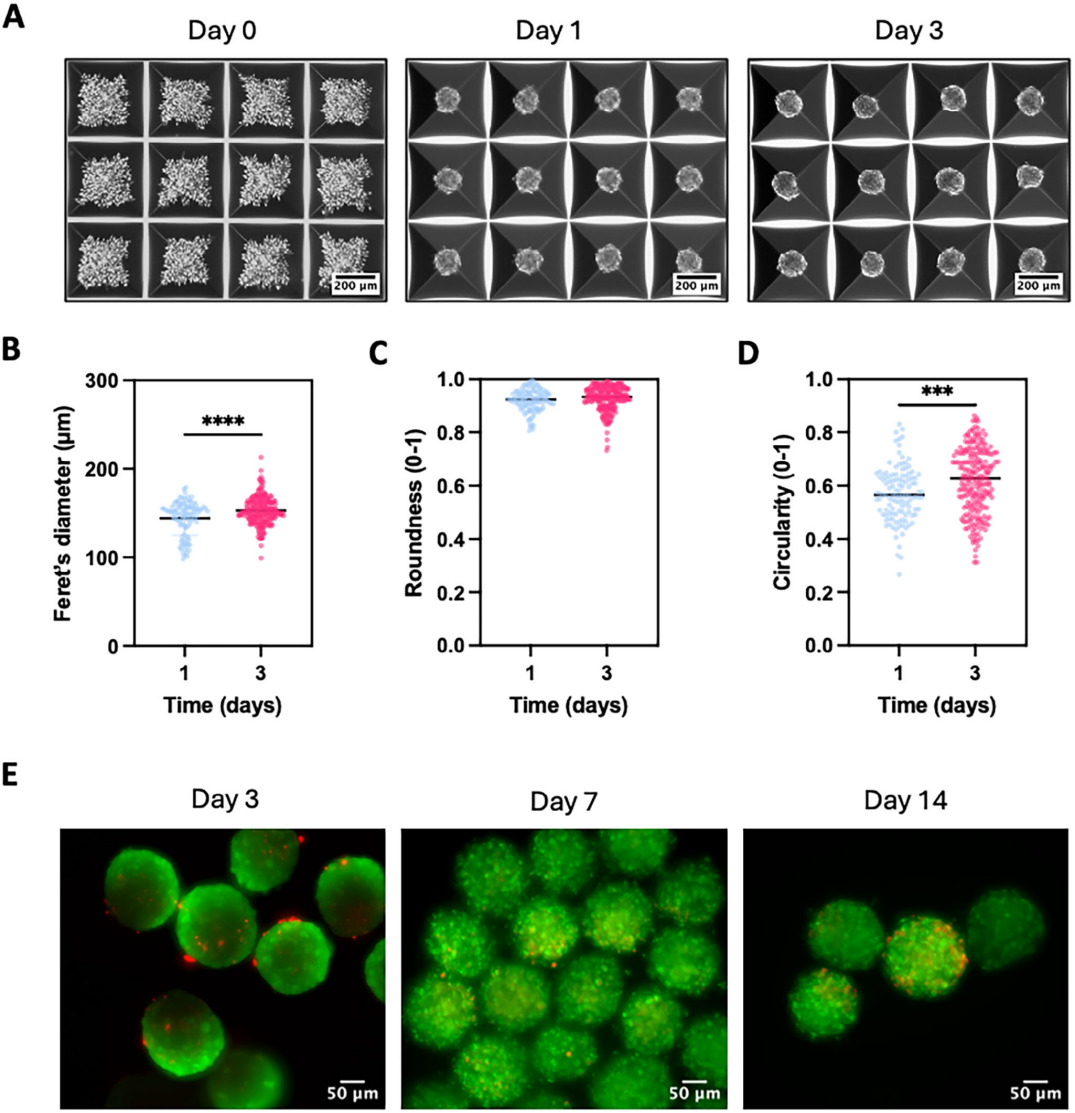
Morphology and viability of ACPC spheroids over time in AggreWell 400 plates. (A) Brightfield images of ACPC spheroid formation over 3 d in chondrogenic differentiation culture. Measurement of (B) Feret's diameter, (C) roundness, and (D) circularity of the spheroids on day 1 and day 3. (E) Representative live/dead staining images to assess spheroid viability on days 3, 7, and 14 post‐seeding. Statistical significance is indicated as *** (*p* < 0.001) or **** (*p* < 0.0001), with *n* > 100 spheroids from *n* = 3 biological replicates.

In this study, equine ACPC spheroids consisting of ≈1000 cells were formed in a high‐throughput microwell system and cultured in chondrogenic differentiation medium for up to 3 d before harvesting, maintaining an early‐stage maturation state. This state is expected to promote spheroid fusion and re‐organization into neo‐cartilage tissue, in line with recent findings [57]. The morphology and size of the ACPC spheroids were observed during those 3 d in chondrogenic differentiation culture (Figure 3A). As indicators of cell‐cell adhesion, uniformity of cell distribution, and overall structural integrity, both the spheroid roundness and circularity were quantified [58, 59]. Our findings show that the spheroid diameter slightly increased over the culture period, reaching approximately 150 µm on day 3 (Figure 3B). Across all three donors used in this study, the average spheroid diameter on day 3 did not differ significantly (Figure S7). On day 1, the cells formed well‐defined, round spheroids (Figure 3C). Over the culture period, spheroid circularity improved significantly, indicating enhanced cell–cell interactions (Figure 3D). Additionally, to evaluate their viability over a longer culturing period inside the microwell system, live/dead staining was performed on days 3, 7, and 14 post‐seeding of the ACPCs into the microwells (Figure 3E). The spheroids remained highly viable throughout the culture period, with only a minimal presence of dead cells and no evidence of necrotic core formation.

### 2.4 Hydrogel Cytocompatibility and Matrix Deposition by Encapsulated ACPC Spheroids

**FIGURE 4:**
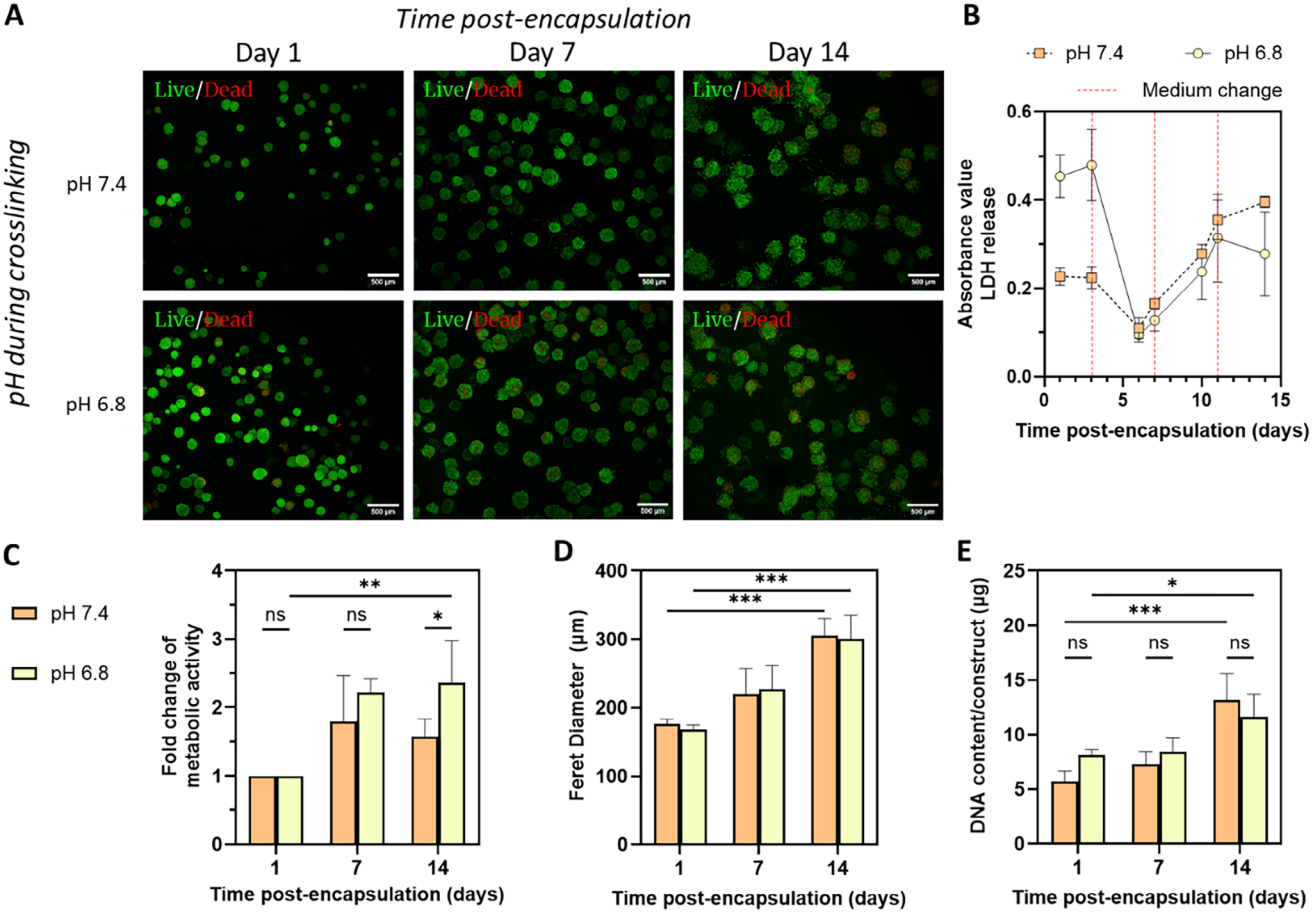
Encapsulation of eqACPC spheroids shows good cytocompatibility of HA/Gelatin/PEG‐based DA hydrogels crosslinked in pH‐altered medium. (A) Confocal microscopy images of encapsulated ACPC spheroids with calcein AM (live)/propidium iodide (dead) staining at various timepoints after crosslinking at either pH 7.4 or pH 6.8. All samples were treated equally after 4 h of crosslinking. Scale bars represent 500 µm. (B) LDH release, (C) Metabolic activity, (D) Feret diameter and (E) DNA content of encapsulated ACPC spheroids over time after crosslinking. In all graphs, values represent the average ± st.dev. of *n* = 3 technical replicates, donor #3. Statistical significance in bar graphs is denoted as * (*p* < 0.05), ** (*p* < 0.01), *** (*p* < 0.001) or **** (*p* < 0.0001).

Further experiments were conducted to assess the 14‐day cell viability, proliferation, and metabolic activity of ACPC spheroids encapsulated in DA hydrogels at pH 7.4 or 6.8. Herein, it is important to note that subsequent culturing after polymer crosslinking was the same for all conditions, using the chondrogenic differentiation medium formulation as described within the method section. ACPC spheroids were harvested 3 d post‐seeding and were then encapsulated at a pH of either 7.4 or 6.8, and polymers were crosslinked for 4 h. On days 1, 7, and 14 post‐encapsulation, the overall cell viability of the ACPC spheroids was assessed using fluorescent live/dead staining. In line with the results of 2D‐cultured ACPCs mentioned earlier (Figure S6), the viability of the ACPC spheroids was not affected by the lower pH value during the first 4 h incubation. On day 1, 7, and 14 alike, cell viability visually appears to be high for all three ACPC donors (Figure 4A for donor #3, and Figures S8 and S9 for donors #2 and #1, respectively) Slightly different results were obtained from a quantitative lactate dehydrogenase (LDH) assay, conducted over the first 14 d after spheroid encapsulation. As visualized in Figure 4B, spheroids release significantly more LDH on day 1 when they are encapsulated in the hydrogel at pH 6.8 compared to pH 7.4, indicating increased cell stress or apoptosis after encapsulation in the lower pH condition. However, there is some degree of donor‐to‐donor variability, as this phenomenon was not seen for another donor (Figure S8). Of note, the released LDH on day 1 in the pH 6.8 condition still only corresponds to less than a third of the amount of LDH released when ACPC spheroids were lysed on the day of harvesting, indicating that the viability of the spheroids was preserved despite initial stress (Figure S10 and Figure 4B), as also seen previously in literature [60]. Additionally, after this initial spike in LDH release on the first day post‐encapsulation, the spheroids behave similar in both conditions afterwards, as indicated by the equal levels of LDH measured. The rise in LDH that is released at later timepoints can be explained by the notion that the ACPCs continue to proliferate during culturing. Even if the relative LDH release per cell does not change, the total concentration of LDH increases. Noteworthy is also that for both conditions, LDH levels remain lower than the values measured on day 1 and 3 at all subsequent timepoints, demonstrating the gel itself is not toxic, but the process of encapsulation can cause some stress.

Additionally, in terms of metabolic activity, spheroid diameter, and DNA content, gels crosslinked at pH 6.8 perform equally good as gels crosslinked at pH 7.4 in the first 14 d (Figure 4C–E). For both conditions, these aforementioned parameters show a significant increase over time, thereby indicating proliferation of the ACPCs within the spheroids. When looking at the metabolic activity and DNA content as a measure of total cell content in the gels after day 21, a clear difference in favor of the pH 6.8 condition is visible. Due to significant hydrogel degradation of the formulations formed at pH 7.4, the metabolic activity and DNA content of these samples could not always be measured after day 21 (Figure S11). This highlights the importance of crosslinking at pH 6.8 when aiming for cell‐encapsulation for at least 4 weeks.

Because of the lacking stability of the formulation crosslinked at pH 7.4, as well as an initial Young's Modulus in the range of healthy native cartilage pericellular matrix for the formulation crosslinked at pH 6.8 [11], further studies were solely conducted on the 6.5% TPC DA hydrogel crosslinked at pH 6.8. For the purpose of cartilage engineering, it is essential that the hydrogel allows differentiation of encapsulated ACPCs and subsequent cartilaginous ECM deposition. Chondrogenic differentiation of encapsulated ACPC spheroids was assessed through biochemical assays for GAG and DNA content, as well as histological examination after 28 d of culture. Spheroid‐free DA hydrogels were used as controls to determine the background levels in all assays.

**FIGURE 5:**
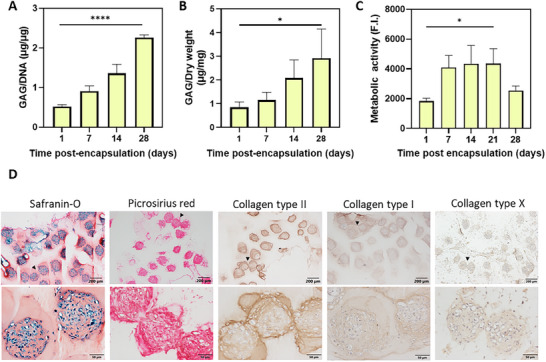
Chondrogenesis of ACPC spheroids following encapsulation in DA hydrogels crosslinked at pH 6.8. (A) Quantitative analysis of GAG/DNA and (B) GAG/dry weight. In all graphs, values represent the average ± st.dev. of *n* = 3 technical replicates, donor #3. Statistical significance in bar graphs is denoted as * (*p* < 0.05), ** (*p* < 0.01), *** (*p* < 0.001), or **** (*p* < 0.0001). (C) Quantification of cell metabolic activity using the Alamar Blue assay. (D) Histological evaluation of the constructs after 28 d in culture. Representative images of safranin‐O/fast‐green, picrosirius red, collagen type II, collagen type I, and collagen type X stainings. Black arrows in the lower magnification images (top row) indicate the regions shown at higher magnification (bottom row). Donor #3 is shown in this figure as a representative for all three donors.

At various culturing times, spheroid‐laden gels were collected and digested prior to quantification of the GAG deposition. As depicted in Figure 5A,B, the amount of GAGs inside the constructs increased significantly already in the first 14 d, demonstrating that the ACPC spheroids were undergoing chondrogenic differentiation and actively deposited ECM. Additionally, the observed plateau in metabolic activity from day 7 onwards may indicate reduced cell proliferation accompanied by increased chondrogenic differentiation (Figure 5C). To further characterize the composition of the deposited ECM and evaluate its appearance over time, hydrogels were fixed and cryosectioned at different time points. Positive safranin‐O and picrosirius red staining, which stain for the proteoglycans and total collagen, respectively, were visible in samples from all three donors, confirming the deposition of cartilaginous matrix from the hydrogel‐embedded ACPC spheroids (Figure 5D and Figure S12). By day 28, the spheroids remained visible, and matrix was deposited both pericellular and around the spheroids as a whole. Immunostaining revealed the presence of both collagen type II and I (Figure 5D), with some variability observed among donors (Figure S13). Collagen type II, a key component of the cartilage typically associated with the formation of hyaline cartilage matrix, was distributed throughout the construct, with more intense staining around the spheroids. In contrast, collagen type I, which is usually more prevalent in fibrocartilage, was more intensely present in parts of the hydrogels where ACPCs exhibited a more cellular migratory behavior as opposed to spheroid fusion. This migratory behavior was already seen in the live/dead staining for multiple donors on day 14 (Figure S14), where cells tended to migrate toward neighboring spheroids without achieving complete fusion of spheroids. In those parts, a faint presence of collagen type X was also detected, though at low levels (Figure 5D and Figure S13). Overall, collagen type X expression remained low throughout the construct, aligning with previous reports showing that ACPCs maintain a non‐hypertrophic phenotype following chondrogenic differentiation and hydrogel encapsulation [53, 61, 62]. This migratory cell behavior has also been observed in human ACPC constructs, where the ACPCs from neighboring spheroids exhibited increased intermixing within the fused structures after 21 d in chondrogenic differentiation culture [63]. Taken together, we show clear evidence that the hydrogel itself provides a supportive surrounding for the successful chondrogenic differentiation of ACPC spheroids.

### 2.5 Effect of Inter‐Spheroid Distance on Spheroid Fusion and Collagen II Production

The fusion of spheroids is essential to form unified, larger tissue engineered constructs. During that process two or more spheroids coalesce, and the cells subsequently migrate to make new connections with the neighboring spheroids. While the fusion phenomenon largely depends on inter‐spheroid cell migration, the distance between the spheroids and their ability to interact is crucial [64, 65, 66]. Cell behavior and spheroid fusion efficiency are also highly affected by the properties of the surrounding hydrogel, particularly its viscoelasticity and degradation rate [49, 65]. The migratory pattern without subsequent spheroid fusion that is associated with collagen I deposition, as seen in Figure 5, might have resulted from the large initial distance between spheroids after encapsulation. Using known parameters such as the volume of a single spheroid; the number of spheroids embedded per hydrogel; and the swelling capacity of the hydrogel, it could be calculated that the average mutual distance between spheroid edges was over 500 µm on day 1, after initial swelling of the construct. Based on literature, there is evidence that the distance between spheroids of different cell types modulates the fusion dynamics, showing primarily fusogenic behavior at mutual distances of 100 µm or less, but largely migratory behavior at distances larger than 400 µm apart [64, 65]. In cartilage tissue engineering, densely packed spheroid arrangements are commonly used to facilitate contact, promote fusion, and enhance chondrogenic tissue maturation [49, 54, 57, 63, 67].

We hypothesized that lowering the initial inter‐spheroid distance would enhance spheroid fusion and induce a more pronounced collagen type II production, while lowering the collagen type I deposition. By increasing the spheroid concentration by over a factor of 10, we could achieve average inter‐spheroid distances of 100 to 150 µm on the first day of culture.

**FIGURE 6:**
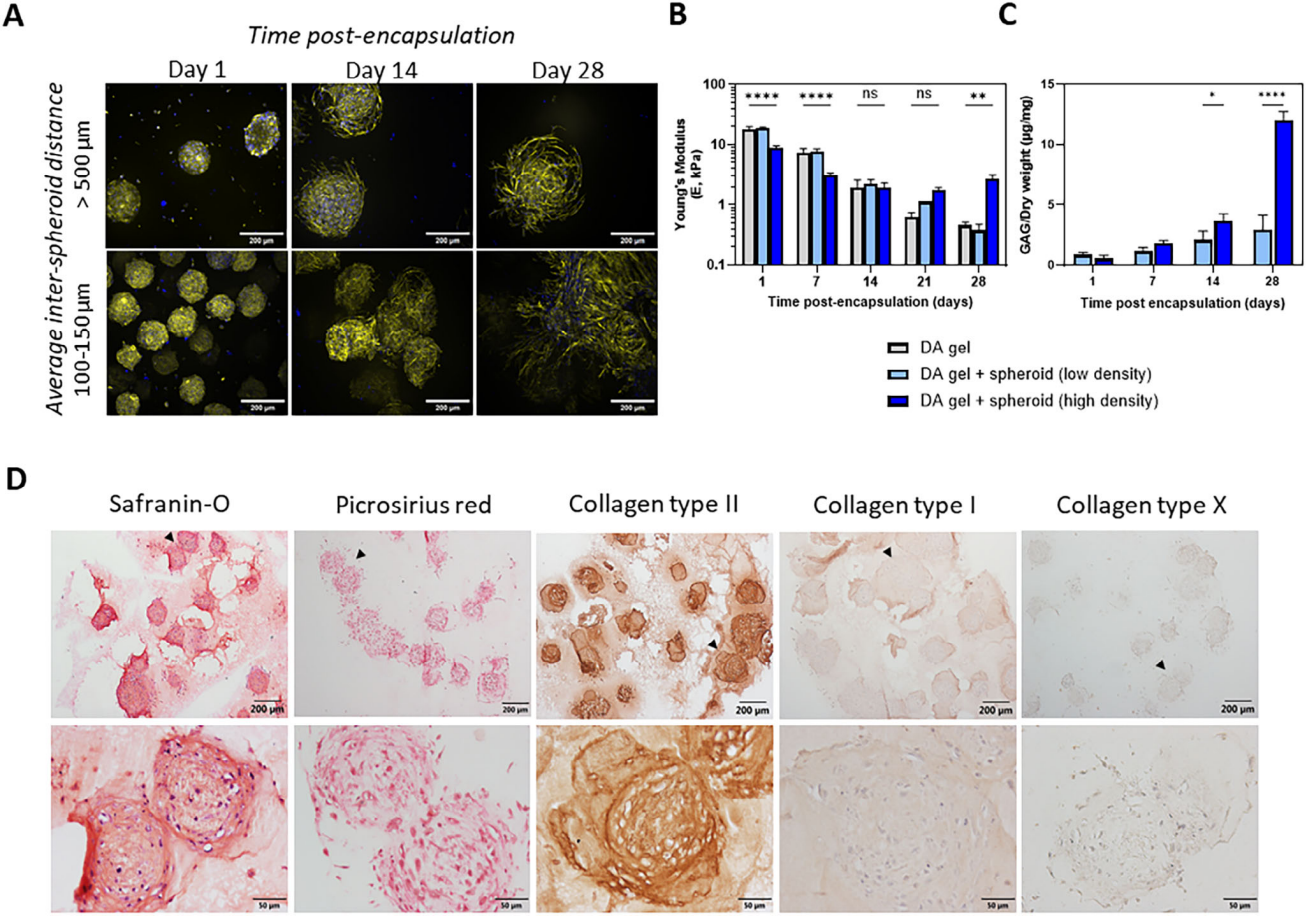
Lowering the initial inter‐spheroid distance promotes spheroid fusion and increases cartilaginous ECM production. (A) Confocal microscopy images of encapsulated ACPC spheroids with Hoechst (cell nucleus, blue)/Phalloidin‐488 (Actin, yellow) staining at various timepoints during culture, with spheroids being on average either more than 500 µm (donor #2) or 100 to 150 µm (donor #3) apart after initial swelling. Scale bars represent 200 µm, maximum projection images. (B) Evolution of construct stiffnesses over a culturing period of 28 d, as measured by the Young's modulus on days 1, 7, 14, 21, and 28. (C) Comparative analysis of GAG/Dry weight for hydrogels with low and high concentrations of encapsulated spheroids over 28 d. Data from Figure 5B were re‐plotted here for direct comparison. In all graphs, values represent the average ± st. dev. Statistical significance in bar graphs is denoted as * (*p* < 0.05), ** (*p* < 0.01), *** (*p* < 0.001), or **** (*p* < 0.0001). (D) Histological evaluation of the constructs with spheroids encapsulated at an average inter‐spheroid distance of 100 to 150 µm apart after 28 d in culture. Representative images of safranin‐O/fast‐green, picrosirius red, collagen type II, collagen type I, and collagen type X stainings. Black arrows in the lower magnification images (top row) indicate the regions shown at higher magnification (bottom row).

First, we evaluated whether this difference in initial inter‐spheroid distance influenced the spheroid morphology during culturing. From staining the cell nuclei and actin filaments, we observed that at a distance of over 500 µm, spheroids mostly develop a sprouting morphology, in which the core remains densely packed, but the cells on the edge seem to reach outward (Figure 6A). At an average inter‐spheroid distance of less than 150 µm, however, the occurrences of observed spheroid fusion are much higher.

We then monitored whether the increased spheroid content and associated fusion had an effect on the mechanical properties of the construct. Once per week, the Young's modulus of spheroid‐free and spheroid‐laden hydrogels was measured. Herein, we made the distinction between embedding spheroids at a low or high density, corresponding to a large (>500 µm) or small (100–150 µm) inter‐spheroid distance (Figure 6B). As expected, the stiffness on day 1 did not differ between the DA gel without and with low density of spheroids, indicating the hydrogel is responsible for the bulk construct properties. However, when the spheroids were encapsulated at high density, there was a decrease in hydrogel stiffness. This may be attributed to either suboptimal hydrogel crosslinking caused by the volume occupied by the spheroids (19.5% of total hydrogel volume for high density versus 1.8% for low density spheroids), or to slight degradation of the hydrogel already on the first day, due to hyaluronidases and collagenases produced by encapsulated cells. As time in culture progresses, the hydrogel constructs soften while swelling and degrading. The softening pattern is the same for the spheroid‐free DA hydrogel and the hydrogel encapsulated with spheroids at a low density, eventually reaching a Young's modulus of less than 1 kPa after 28 d in culture. Interestingly, this is not the case for the DA gel with spheroids encapsulated at a high density: the measured Young's modulus plateaus after 7 to 14 d in culture, and even increases again after 28 d, to a final Young's modulus of more than 2.5 kPa.

To determine whether increased ECM deposition could be a reason for the enhanced mechanical properties observed after 28 d, biochemical quantification of the GAG content (Figure 6C; Table S1) and histological evaluation of the ECM deposition (Figure 6D; Figure S15A) were performed. On day 28, histological and immunohistochemical analysis revealed increased deposition of GAGs and collagen type II, compared to low spheroid density hydrogel constructs (Figure 5D). Quantification of the relative ratio of collagen type II to collagen type I positive areas (Figure S15B) further confirmed this observation. Where in low spheroid density constructs, collagen type II and I were found to be relatively comparable in abundance, the relative ratio of collagen type II to collagen type I was highly increased upon lowering the average distance between spheroids at higher spheroid density. It should be noted that this analysis was based on thin histological sections and, therefore, may not capture the entire 3D construct. While spheroid fusion was more apparent at the higher concentration of spheroids, individual spheroid units were still observed throughout the hydrogel. Over 4 weeks of chondrogenic culture, constructs containing encapsulated spheroids at a high density accumulated approximately fourfold higher GAG content per dry weight compared to the low‐spheroid density hydrogel constructs on the same day (Figure 5B,C). To conclude, spheroid density within the hydrogel, and thus inter‐spheroid distance, influenced cell behavior, with higher densities promoting spheroid fusion and enhanced ECM deposition.

### 2.6 Reinforcement of Hydrogels with Melt Electrowritten Scaffolds

We have demonstrated that the 6.5% DA hydrogel crosslinked at pH 6.8 is cytocompatible and its mechanical properties support cartilaginous ECM deposition and spheroid fusion. However, while the initial compressive modulus of the hydrogel is similar to the lower end of the range of the stiffness of the PCM (0.02–0.2 MPa), it lacks the compressive modulus of native cartilage tissue. Depending on the animal species, it can reach up to 4 MPa, though ranges of 0.1 to 1.6 MPa have been reported for humans [11, 41, 68]. To be considered for cartilage implantation, the compressive modulus of the hydrogel needs to be increased to be able to withstand the forces in e.g. a human knee. MEW scaffolds have often been used to reinforce covalently crosslinked hydrogels, as the microfibers in the mesh tend to have a synergistic effect on the mechanical properties of the hydrogel and MEW scaffold. This is particularly interesting for cartilage engineering, as MEW scaffolds have been shown to effectively bridge soft‐to‐hard interfaces such as the cartilage‐bone interface [69], resulting in osteochondral implants that are capable of withstanding the in vivo environment [68]. Considering that macroscopic porous PCL MEW scaffolds alone are softer than the hydrogels themselves (1.1–15.2 kPa depending on the scaffold porosity) [44], this reinforcement can be explained by multiple other mechanisms. Primarily, the MEW scaffold increases hydrostatic pressure during compression by limiting the discharge of water from the hydrogel. This mechanical restriction also leads to reduced swelling in diameter in the MEW‐reinforced constructs during the first few hours (9 ± 2% for MEW‐reinforced constructs, 27 ± 2% for non‐reinforced constructs, Figure S16), suggesting that the ability of the hydrogel to absorb additional water is exceeded by the scaffold's resistance to deformation. Simultaneously, the stretching of the fibers during lateral expansion and the prevention of buckling of the stacked fibers by the gel increases the overall macroscopic mechanical stiffness during compressive loading [43, 44]. It has been shown that the density of the MEW scaffold directly affects the reinforcement properties of the hybrid construct [70]. MEW scaffolds have successfully been used with organoids for multiple tissue‐models, including adipose tissue [71] and cartilage tissue [57], highlighting its potential as a reinforcing strategy for soft hydrogels containing organoid‐like structures.

**FIGURE 7:**
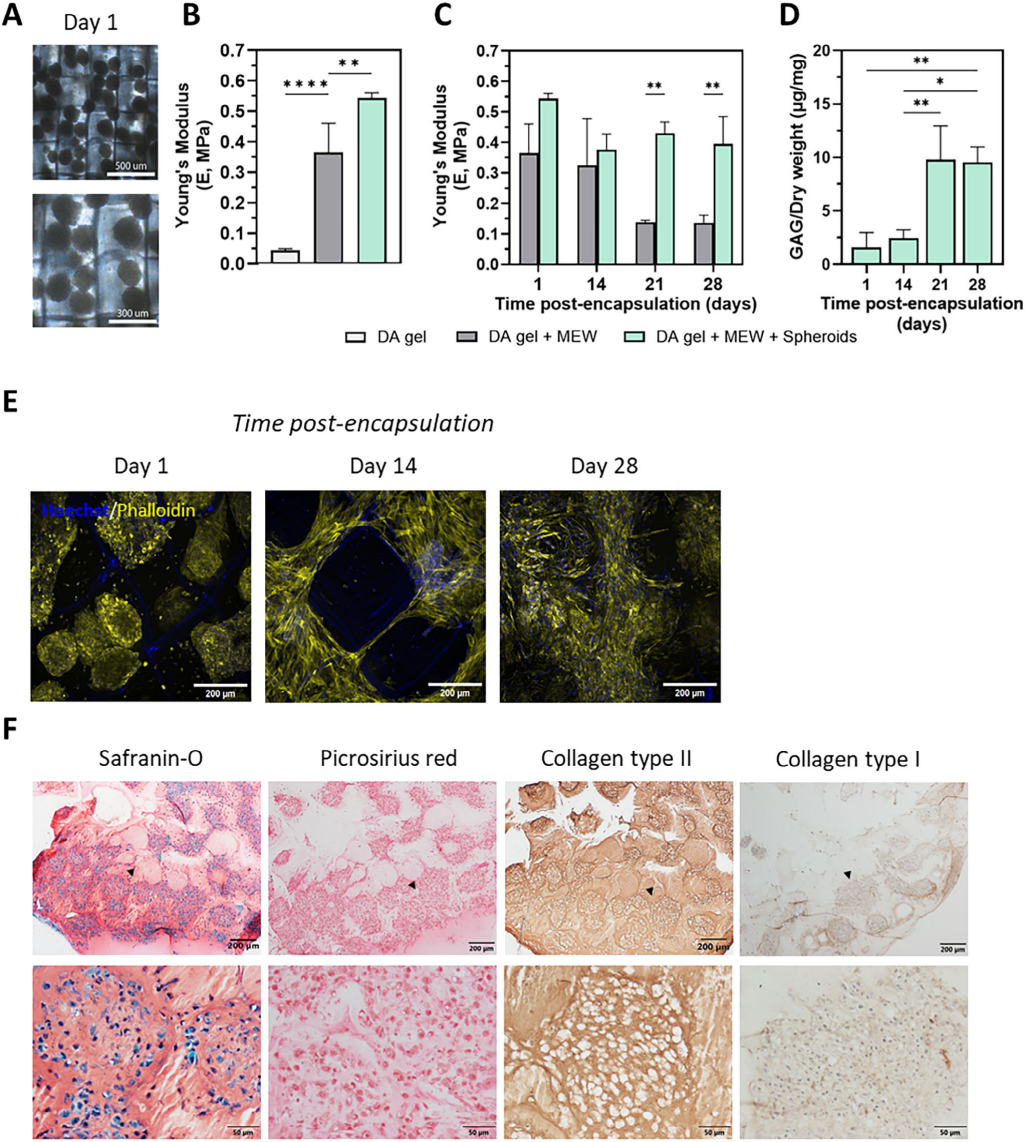
Equine ACPC spheroid‐laden DA hydrogel (6.5% TPC, crosslinked at pH 6.8) constructs can be reinforced with MEW scaffolds (A) Encapsulated eqACPC spheroids are present inside MEW boxes after casting, demonstrating proper placement within the constructs on day 1. (B) The Young's modulus of (reinforced) DA hydrogels and spheroid‐laden DA hydrogels 2 h after preparation on day 0. (C) Evolution of construct stiffnesses over a culturing period of 28 d, as measured by the Young's modulus on days 1, 14, 21 and 28. (D) GAG deposition in MEW reinforced, spheroid laden hydrogels over a culturing period of 28 d. (E) Spheroid morphology inside MEW reinforced hydrogel constructs, as visualized by Hoechst (blue, cell nuclei) / Phalloidin (yellow, actin filaments) staining on confocal microscopy. Scale bars represent 200 µm at 20x magnification. (F) Histological evaluation of the constructs after 28 d in culture. Representative images of safranin‐O/fast‐green, picrosirius red, collagen type II, and collagen type I stainings. Black arrows in the lower magnification images (top row) indicate the regions shown at higher magnification (bottom row).

Casting of the spheroid‐laden gel into the MEW scaffolds was successfully achieved (Figure 7A). The PCL MEW scaffold exhibits an eight‐fold reinforcing effect in spheroid‐free constructs when compressed perpendicular to the plane of the scaffold (from the top side), indicating direction‐dependent mechanical properties (Figure 7B). Interestingly, the reinforcing effect was not only observed upon inclusion of the MEW scaffold; the inclusion of spheroids at high density further enhanced the macroscopic stiffness of the constructs on day 1, resulting in a total 12‐fold stiffness increase. This additional stiffness may be attributed to the spheroids themselves contributing to load‐bearing, thereby further increasing the compressive resistance of the constructs [63].

The spheroid‐laden MEW‐reinforced DA hydrogels were cultured in chondrogenic differentiation medium for 28 d. Periodically, the construct stiffness was tested by assessing the Young's modulus. As was previously seen in non‐reinforced DA hydrogels, the reinforced spheroid‐free DA hydrogel showed construct softening after 21 and 28 d, indicative of the expected hydrogel degradation (Figure 7C). Similar to the results seen in Figure 6b, this pattern was not found in the reinforced spheroid‐laden constructs. Instead of continuous construct softening, the construct stiffness stabilized after an initial decrease between day 1 and day 14 and remained at a value of around 400 kPa up to day 28. When examining GAG deposition relative to dry weight, it is evident that between days 14 and 28, the production of GAGs vastly increased (Figure 7D). We hypothesize that the GAG deposition is partially responsible for the stability of the construct stiffness, and that the spheroids replace the hydrogel matrix as it degrades.

Next, the overall morphology of the spheroids inside the construct during culturing was investigated. Cell nuclei and actin filaments were visualized, showing distinct, separated spheroids inside the MEW boxes on day 1; fusion and migration alongside the MEW mesh on day 14; and a primarily fused construct on day 28 (Figure 7E). Within the individual MEW boxes, the spheroids appear to take up all available space over time. As spheroids were encapsulated into the MEW‐reinforced hydrogel by pipetting and casting, the distribution of spheroids over the construct was extensive, but not completely homogeneous, with many MEW boxes having multiple spheroids, where some had none. In these boxes, we observed that cells attach to the MEW fibers and align with them, slowly filling the boxes over time. Lastly, we evaluated the composition of the deposited ECM on day 28 by means of histological and immunohistochemical analysis (Figure 7F; Figure S17). Consistent with recent findings, the addition of the MEW PCL mesh did not hinder the chondrogenic differentiation and fusion capacity of the encapsulated spheroids [54, 72]. The ACPC spheroids were able to generate ECM rich in GAGs and collagen type II following encapsulation in the reinforced DA hydrogel, as confirmed by intense and spatially consistent collagen type II staining on day 28 (Figure 7F). Additionally, histological analysis revealed that as early as day 14, consistent with confocal microscopy observations, the spheroids had begun to fill the MEW mesh boxes. In several regions, spheroid fusion was evident, with individual spheroid boundaries becoming less distinct and some cells starting to spread through the porous structure of the MEW scaffold (Figure S17). It should be noted that after spheroid encapsulation, the spheroids fuse and mature into a cartilage‐like, yet heterogeneous tissue over time, with a few small cartilage type I‐containing regions. The profile of deposited ECM and low hypertrophy are indeed consistent with the stable cartilage‐like phenotype earlier seen in ACPC‐derived tissue [52, 53]. However, full hyaline cartilage organization with structural collagen alignment is not yet achieved, and thus an avenue for further research. Additionally, the present work is an in vitro optimization and proof‐of‐concept, and future in vivo or ex vivo joint models will be required to confirm long‐term integration and functional performance.

## 3 Conclusion

In this work, we demonstrated that hydrogels crosslinked by dynamic‐covalent Diels‐Alder click chemistry can be tailored for cartilage tissue engineering. As a reversible covalent crosslinking mechanism, the Diels‐Alder reaction provides an ideal combination of stability and reconfigurability, supporting spheroid fusion over culture periods of up to four weeks. Additionally, the absence of catalysts or UV triggers during hydrogel formation represents a key translational advantage over commonly used hydrogels, such as GelMA. Although Diels‐Alder hydrogels were previously reported in literature, we addressed common challenges, including insufficient hydrogel integrity, slow gelation, and rapid degradation by lowering the pH during crosslinking. This way, we developed a hydrogel with a stiffness comparable to the lower end of the stiffness range found in native pericellular matrix in human articular cartilage, while also achieving sufficient stability to support the culture of encapsulated equine ACPC spheroids for at least 28 d. To our knowledge, the fusion of multicellular spheroids and their maturation towards a cartilage‐like tissue inside a hydrogel solely crosslinked by Diels‐Alder chemistry has not been investigated before. We showed that the spheroids remain viable for a culture period of 28 d post‐crosslinking and deposited cartilaginous ECM rich in collagen type II. Herein, a lower average inter‐spheroid distance of 100–150 µm as opposed to >500 µm is important to enhance spheroid fusion and matrix deposition. While the encapsulated spheroids were surrounded by the cell‐friendly DA hydrogel with PCM‐like material properties, reinforcement with a MEW PCL scaffold enhanced the compressive modulus of the entire construct 12‐fold on day 1 and over 100‐fold by day 28 compared to non‐reinforced constructs. This reinforcement improved the potential future applicability of the spheroid‐laden DA hydrogel as a cartilage tissue implant.

## 4 Experimental Section

### 4.1 Materials

#### 4.1.1 Synthesis Reagents

Sodium hyaluronate (Molecular weight 381 kDa and 391 kDa) was purchased from LifeCore BioMedical (Chaska, MN). Gelatin (Gel, porcine, type A, 300 bloom) was kindly provided by Rousselot (Ghent, Belgium), and 4‐arm polyethylene glycol (PEG)‐maleimide (4APM, molecular weight 10 kDa) was bought from JenKem Technology (Plano, TX). Furfurylamine, furfuryl glycidyl ether, 2‐(*N*‐morpholino)ethane sulfonic acid (MES) sodium salt, 4‐(4,6‐dimethoxy‐1,3,5‐triazin‐2‐yl)‐4‐methylmorpholinium chloride (DMTMM) and deuterium oxide were obtained from Sigma‐Aldrich. Ethanol was purchased from Biosolve (Valkenswaard, the Netherlands). The 12–14 kDa molecular weight cut‐off (MWCO) dialysis membranes were obtained from Carl Roth GmbH (Karlsruhe, Germany). All required reagents for the 2,4,6‐trinitrobenzene sulfonic acid (TNBSA) assay were obtained from Thermo Scientific. ε‐Polycaprolactone (PCL) was provided by Corbion (PC‐12, 72 kDa, The Netherlands).

#### 4.1.2 Cell Culture Reagents

Proteinase K, hyaluronidase Type IV‐S and type I‐S, pronase, fibronectin, resazurin sodium salt, Triton‐X‐100, Tween 20, bovine serum albumin (BSA), chondroitin sulphate, Dulbecco's Phosphate Buffered Saline (PBS), Weigert's hematoxylin, Eukitt mounting medium, DAPI and goat serum were all acquired from Sigma‐Aldrich. Quant‐iT PicoGreen dsDNA assay kit was bought from Thermo Scientific. The antibodies mouse‐anti‐collagen type X53 and goat‐anti‐mouse Alexa‐Fluor 488, propidium iodide, Hoechst 33342, Phalloidin‐AlexaFluor‐647 and Phalloidin‐AlexaFluor‐488 were also purchased from Thermo Scientific. Rabbit‐anti‐collagen‐I antibody (EPR7785, ab138492) was obtained from Abcam, and mouse‐anti‐collagen type II was provided by DHSB. Anti‐mouse IgG HRP (P0447) was obtained from Dako, whereas BrightVision+ anti‐rabbit IgG was obtained from VWR. Goat anti‐mouse IgG secondary antibody Alexa Fluor 488 and donkey anti‐rabbit IgG secondary antibody Alexa Fluor 594 were purchased from Invitrogen. Collagenase type‐II was bought from Worthington Biochemical, DAB substrate kit peroxidase was obtained from Agilent Technologies and cytotoxicity detection kitPLUS (LDH) was purchased from Roche. Calcein AM was obtained from Bio‐Connect (Santa Cruz), and Tissue‐Tek Optimal Cutting Temperature (O.C.T.) was acquired from Sakura. Hank's Balanced Salt Solution (HBSS) was purchased from LifeTech. Immu‐Mount was bought from Epredia.

Chondroprogenitor expansion medium consisted of Dulbecco's modified Eagle's medium (DMEM)‐high glucose (31966, Thermo Scientific) supplemented with 10% Fetal bovine serum (FBS, Capricorn‐Scientific), 1% ascorbic acid‐2‐phosphate (ASAP, Sigma‐Aldrich), 1% penicillin and streptomycin (P/S, Gibco), 1% MEM non‐essential amino acids (NEAA, Gibco) solution, and 5 ng mL^−1^ basic fibroblast growth factor (bFGF, R&D Systems). Chondrogenic differentiation medium contained DMEM‐high glucose, 1% ASAP, 1% P/S, 1% ITS+ premix (Corning), 10 mm 4‐(2‐hydroxyethyl)‐1‐piperazineethanesulfonic acid (HEPES, Thermo Scientific), 4 µg mL^−1^ dexamethasone (Sigma‐Aldrich) and 1 µg mL^−1^ transforming growth factor β1 (TGF‐β1, PeproTech). DMEM‐High Glucose supplemented with 10 mm HEPES, 1% P/S and 1% ASAP was considered relevant cell culture medium imitation. pH‐adapted medium was prepared from DMEM‐high glucose without sodium bicarbonate and sodium pyruvate (Sigma‐Aldrich), supplemented with 20 mm HEPES and 20 mm piperazine‐*N,N*′‐bis(2‐ethanesulfonic acid) (PIPES) buffer (Sigma‐Aldrich), 3% antibiotic/antimycotic (Sigma‐Aldrich, to a concentration of 300 units mL^−1^ penicillin, 0.3 mg mL^−1^ streptomycin, 0.075 µg mL^−1^ amphotericin B), and HCl or NaOH to adapt the pH.

### 4.2 Methods

#### 4.2.1 Functionalization of Hyaluronic Acid with Furfurylamine (HAFU)

Furan‐modified HA (HAFU) was prepared by reacting the HA acid groups with furfurylamine according to a previously reported method with minor modficiations [26]. Briefly, sodium hyaluronate (381 kDa; 2.5 g; 6.3 mmol disaccharide units, 1 eq) was dissolved in 300 mL MES buffer (100 mm, pH 5.5), to which DMTMM was added (4.25 g, 15.4 mmol, 2.4 eq) and stirred for 1 h at RT. Subsequently, furfurylamine was added in a dropwise manner (1.1 mL, 12.7 mmol, 2 eq), and the mixture was allowed to react for 48 h at room temperature (RT) under vigorous stirring. The reaction was terminated by raising the pH to 7.0 with 4 m NaOH. HAFU was precipitated in ethanol using a 1:7.5 ratio of reaction mixture H_2_O to ethanol and washed three times with ethanol prior to vacuum‐drying of the precipitate to obtain HAFU (2.2 g) as a white powder. Two batches of HAFU were prepared and combined after drying, each obtained with a yield between 74 and 78%. The polymer was characterized with ^1^H‐NMR spectroscopy in D_2_O (Agilent 400 MHz NMR spectrometer, Agilent Technologies, CA, Figure S1). Data analysis was conducted using MestReNova version 14.2, where the residual solvent peak (4.79 ppm for H_2_O) was used to calibrate the chemical shifts. The ratio of the integrals of the *N*‐acetyl glucosamine peak on the HA‐backbone (2.1 ppm) and the aromatic furan peaks (7.5 and 6.4 ppm) was considered as the degree of functionalization (65% DF). ^1^H‐NMR δ (D_2_O, ppm): 7.5 (OCH; 1H; d), 6.4 (CHCH; 2H; d), 4.10‐3.0 (protons of HA‐backbone; m), 2.1 (NHCOCH_3_; 3H; s). The synthesis to modify HA 391 kDa with furfurylamine was conducted in a similar manner (60% DF, 5.3 g, 93% yield, Figure S16). HAFU 381 kDa was used for all experiments up until section 2.4. After this, HAFU 391 kDa was used.

#### 4.2.2 Functionalization of Gelatin with Furfuryl Glycidyl Ether (GelFU)

Furan‐modified gelatin (GelFU) was obtained by reacting the primary amines of lysine residues present on gelatin with furfuryl glycidyl ether, following a protocol described by Garcia‐Astrain et al. [73]. Gelatin (2.5 g) was dissolved in 500 mL demineralized water at 40°C, after which the pH was adjusted to 11 using 0.5 m aqueous NaOH solution. The solution was cooled in an ice bath, followed by dropwise addition of furfuryl glycidyl ether (1.0 mL, 7.3 mmol). Next, the reaction mixture was stirred at 55°C for 24 h under a nitrogen atmosphere, before neutralization of the solution to pH 7.0 by addition of 0.1 m HCl. The mixture was dialyzed against H_2_O for 48 h using a 12–14 kDa MWCO dialysis membrane. After freeze‐drying, GelFU (2.2 g) was obtained as a fluffy white powder with a yield of 87%. The presence of furan groups on gelatin was confirmed with ^1^H‐NMR spectroscopy in D_2_O (7.6 and 6.5 ppm for the aromatic furan peaks), but due to a lack of a reference signal, the degree of functionalization could not be determined by ^1^H‐NMR (Figure S3). Therefore, a TNBSA assay was conducted according to the manufacturer's protocol to determine the amount of unreacted amines after functionalization with furan, using unmodified gelatin to determine the total amount of lysine residues (91.3% DF).

#### 4.2.3 Preparation of Hydrogel Disks

Cylindrically shaped HAFU/GelFU/4APM hydrogels were prepared in polydimethylsiloxane (PDMS) molds (diameter × height of 6 mm × 2 mm or 5 mm × 3 mm). Specifically, HAFU and GelFU were weighed in a 4:1 mass ratio and co‐dissolved overnight at 37°C in the desired medium (either PBS or relevant culture medium imitation (see Section 4.1.2.), or pH‐adapted medium (see Section 4.1.2.)). 4APM was dissolved separately and directly prior to use. The polymers were mixed in a molar ratio of 5:1 furan to maleimide (calculated by amount of furan groups on HAFU and GelFU) as optimized by Ilochonwu et al. [26], to obtain a total polymer content of 3.5, 4.5, 5.5 or 6.5 wt% (see Figure 1C). After mixing, hydrogels were allowed to crosslink in a humidified incubator at 37°C and 5% CO_2_ for 4 h.

#### 4.2.4 Rheological Characterization

The rheological properties of different hydrogel compositions (3.5, 4.5, 5.5, or 6.5 wt% in PBS, cell culture medium or pH‐adjusted medium) were investigated using a Discovery HR‐2 Rheometer (TA Instruments, Etten‐Leur, The Netherlands), equipped with a Peltier plate to regulate temperature and a solvent trap to minimize solvent evaporation. All samples were measured employing a 20 mm diameter aluminum plate‐plate geometry with a static gap of 300 µm. Hydrogel precursor solutions were prepared as described above, and 108 µL sample was deposited under the geometry at 8°C before measurements at 37°C. Data was processed with TRIOS Software version 5.1. Experiments were conducted at strain values within the linear viscoelastic region (LVR, Figure S3). Gelation kinetics were then evaluated by measuring the storage (G′) and loss (G″) moduli at 37°C upon constant oscillation at a frequency of 1 Hz and strain of 1.0%.

#### 4.2.5 Dynamic Mechanical Analysis (DMA)

DMA measurements were performed on a DMA Q800 Dynamic Mechanical Analyzer (TA instruments, Etten‐Leur, The Netherlands), in two different modes. The Young's modulus (*E*) of hydrogels formed in pH‐adjusted medium was determined using controlled force compression. Fully crosslinked hydrogels (5 mm × 3 mm) were prepared as described in Section 4.2.3., taken out of the PDMS molds and left in relevant culture medium imitation (see Section 4.1.2.) at 37°C and 5% CO_2_ for 2 h prior to measuring. Dimensions of the cylinder were measured, and the hydrogel was placed between the parallel compression plates (upper plate 20 mm, lower plate 45 mm diameter). A force ramp was applied between 0.001 and 0.2 N at a rate of 0.02 N min^−1^ at RT. Data was processed with TA Universal Analysis software, where the slope of the linear section of the stress‐strain curve was used to calculate the Young's modulus. For comparing the development of the Young's modulus over a period of 28 d between empty and spheroid‐laden hydrogels, hydrogels were prepared in a Teflon mold (6 mm × 2 mm) and left in chondrocyte differentiation medium for approximately 24 h prior to measurements on day 1. For each timepoint during the experiment, three different gels were used.

To identify the compressive Young's modulus over time in cell‐laden constructs, MEW‐reinforced disks (6 mm × 1 mm) were formed in PDMS molds and tested at RT. A preload of 0.001 N was applied to ensure full contact with the sample and to measure its height. Subsequently, a strain ramp of ‐20% min^−1^ was applied, measuring from 0 to ‐30%. Stress was recorded simultaneously at a rate of two measurements per second. The slope of the linear region of the stress–strain curve, starting after 10% strain, was used to determine the compressive Young's modulus. Samples were measured at day 1, 14, 21, and 28 with three samples per timepoint.

#### 4.2.6 Swelling and Degradation

Cylindrical crosslinked hydrogels (6 mm × 2 mm) were prepared as described in Section 4.2.3. and placed in a pre‐weighed 2 mL Eppendorf tube to perform swelling and degradation tests. The weight of the hydrogel was recorded right after crosslinking (W_0_), after which 1 mL of relevant culture medium imitation (Section 4.1.2.) was added. Samples were stored stationary at 37°C. At regular intervals, the medium was removed from the tube and the weight of the hydrogel was determined (*W*_x_). Successively, 1 mL of fresh medium was added for further incubation at 37°C. The weight ratio was calculated as the weight at a specific point in time (W_x_) divided by the initial hydrogel weight: *W*_x_/*W*_0_.

#### 4.2.7 Cell Isolation and Culture

Equine articular cartilage progenitor cells (ACPCs) were isolated from macroscopically healthy metacarpophalangeal joints of two deceased skeletally mature adult equine donors (donor #2 and #3) and one juvenile horse (donor #1), following previously established methods [53, 74]. The donors had been donated to science by their owners, and all procedures were conducted in accordance with the guidelines of the Ethical and Animal Welfare body of Utrecht University. Briefly, the cartilage tissue was minced and digested in 0.2% pronase for 2 h at 37°C with agitation, followed by an overnight incubation in a 0.075% collagenase type II solution. The tissue digest was filtered using a 70 µm strainer, and the isolated single cells were washed, suspended in serum‐free DMEM and subjected to a fibronectin adhesion assay. The cells were plated in fibronectin‐coated tissue culture flasks at a density of 500 cells cm^−2^, followed by a 20 min incubation at 37°C. Next, non‐adherent cells were washed away with PBS, and the attached cells were cultured in chondroprogenitor expansion medium up to passage 4.

#### 4.2.8 Effect of Medium pH on Cytocompatibility for Equine Articular Cartilage Progenitor Cells

To address whether 4 h incubation at a pH value other than 7.4 during hydrogel crosslinking affects the metabolic activity of encapsulated cells, single equine articular cartilage progenitor cells (eqACPCs) were seeded at 7.500 cells per well in an adhering 96‐well plate and allowed to settle overnight in a humidified incubator at 37°C and 5% CO_2_. Next, medium was removed and replaced with pH‐adjusted medium with FBS (DMEM‐High Glucose + 10 mm HEPES + 10% FBS + 3% P/S, pH‐adjusted to either 4.0, 5.0, 5.5, 6.0, 6.5, 7.0, 7.5 or 8.0 with sterile‐filtered 6 m HCl or 1 m NaOH in demineralized water), before incubation at 37°C for 4 h. pH‐adjusted medium was aspirated and replaced with chondrogenic differentiation medium. A Cell Titre 96 assay was conducted 24 h post incubation, according to the manufacturer's protocol. After 4 h incubation with the Celltiter 96 reagent, absorbance was recorded at 490 nm on a FluoStar Optima spectrometer (BMG Labtech). All conditions were normalized against the absorbance at pH 7.4 (100%).

#### 4.2.9 ACPC Spheroid Formation and Culture

To generate multicellular spheroids with dimensions of ≈150 µm, eqACPCs (passage 3) were suspended in chondrogenic differentiation medium and seeded in a six‐well microwell plate (Aggrewell 400, Stemcell Technology), following the manufacturer's instructions. Each spheroid contained approximately 1000 cells. The spheroid diameter, roundness (4 × ([area]/ [π (major axis^2^)]), and circularity (4π × [area]/[perimeter^2^]) were quantified after 24 and 72 h post‐seeding using bright‐field microscopy and analyzed with ImageJ. After a 3 d culture period, the spheroids were harvested for gel encapsulation. By gently pipetting up and down 2–3 times per well and using a 40 µm cell strainer, the spheroids could be collected. To prevent attachment of the ACPC spheroids, all plasticware was pre‐coated with 1% BSA in PBS prior to use.

#### 4.2.10 Spheroid Encapsulation

To evaluate the cytocompatibility of the Diels‐Alder hydrogels, 3 day old ≈1000 cell eqACPC spheroids were encapsulated into bulk hydrogels at a concentration of 10.000 spheroids mL^−1^ hydrogel. Typically, hydrogels were prepared as described in Section 4.2.3., combining concentrated spheroid solution with the HAFU/GelFU mixture before adding the 4APM solution. This solution was first incubated at 37°C for 10 min to kickstart the gelation process, and prevent spheroids from sinking to one side of the gel before full gelation can be realized. After additional homogenization, the hydrogel disks were prepared using autoclaved PDMS molds (6 mm diameter, 2 mm height). After a total of 4 h of crosslinking at 37°C, gels were taken out of the mold and placed in a 24‐well suspension plate with 1 mL chondrogenic differentiation medium per gel. Constructs were maintained in a humidified environment at 37°C and 5% CO_2_, with biweekly medium replacements. For all spheroid encapsulation experiments at low spheroid concentration (10.000 spheroids mL^−1^ hydrogel, > 500 µm inter‐spheroid distance), three eqACPC donors were used (donor #1, #2 and #3), of which donor #3 is depicted in the main text as the representative donor. For all spheroid encapsulation experiments at high spheroid concentration (105.000 spheroids mL^−1^ hydrogel solution, 100–150 µm inter‐spheroid distance), donor #3 was used.

#### 4.2.11 Confocal Microscopy

To assess the general viability of the encapsulated spheroids within the construct over time, hydrogels were transferred to a 96‐well imaging plate (Greiner) on days 1, 7, or 14. To visualize the live, metabolically active cells, gels were stained with calcein AM in medium (70 µL, 64 nm) for 3 h at 37°C, followed by propidium iodide (PI, 35 µL, 80 nm) to stain for dead or dying cells directly prior to imaging. Imaging was conducted on a Yokogawa CV7000S spinning disk confocal microscope, using excitation wavelengths of 488 and 561 nm; emission filters of 475–575 and 647–705 nm; and an exposure time of 11 and 250 ms, for Calcein and PI, respectively. Images in the *z*‐axis of the gel were taken every 20, 10 or 5 µm, using a 4, 10 or 20× magnification objective, respectively, which were combined into a single image (maximum projection).

To visualize the cell morphology within spheroids at different time points during the culturing period, hydrogels were washed with PBS for 10 min before fixation in 1 mL 4% paraformaldehyde (PFA) for 1 h. Hydrogels were then washed 3× 5 min with PBS + 0.1% (v/v) Tween‐20, followed by permeabilization of the cells with 0.3% (v/v) triton‐X‐100 in PBS for 90 min at RT while shaking. The solution was removed, and gels were washed 3× for 5 min with 1 mL HBSS. Antigens were blocked in blocking buffer (PBS + 2% (v/v) FBS, 0.5% (w/v) BSA and 0.1% (v/v) Tween‐20) for 90 min at RT while shaking. Actin filaments were stained overnight at RT using a 1:1000 dilution of AlexaFluor‐647‐phalloidin in blocking buffer on a plate shaker. Hydrogels were then washed 3 times for 3 min with HBSS and stained with Hoechst 33342 (1:1000 in blocking buffer) for 1 h. Imaging was conducted on a Yokogawa CV7000S spinning disk confocal microscope, using excitation wavelengths of 405 and 640 nm; emission filters of 400–490 and 647–705 nm; and an exposure time of 500 and 120 ms, for Hoechst and Phalloidin respectively. Images in the z‐axis of the gel were taken every 20 or 10 µm for a total depth of 1400 or 675 µm, using a 4× or 20× magnification objective, respectively, which were combined into a single image (maximum projection).

For all imaging experiments, negative and single stain controls were used on cell‐containing hydrogels to ensure no autofluorescence or carry‐over of emission between channels could be seen at relevant intensities.

#### 4.2.12 Metabolic Activity and Viability

The metabolic activity of encapsulated ACPC spheroids was investigated using an AlamarBlue metabolic activity assay. To prepare 11× AlamarBlue solution, resazurin sodium salt was dissolved in PBS at a concentration of 500 µM and sterilized using a 0.22 µm filter. Hydrogels with cells, along with cell‐free controls, were transferred to a 48‐well plate, where 440 µL of 1× AlamarBlue solution, diluted in chondrogenic differentiation medium, was added to each well. Following a 4 h incubation in a humidified incubator at 37°C and 5% CO_2_, 100 µL of the supernatant was transferred to a black flat‐bottom 96‐well plate (Greiner), and fluorescence was detected at 530/600 nm *λ*_ex_/*λ*_em_. Hydrogels were placed back into fresh medium and maintained for the entire culturing period.

To further evaluate the spheroid viability within the hydrogels, a cytosolic lactate dehydrogenase (LDH) assay was performed using the Cytotoxicity detection kit^PLUS^. LDH is released from the cytosol of damaged or lysed cells into the surrounding medium, making it a reliable marker for cytotoxicity and cell membrane integrity. Supernatant medium was collected on days 1, 3, 6, 7, 10, 11, and 14 post‐encapsulation from three technical replicates. As a positive control, an equal number of suspended ACPC spheroids, corresponding to those encapsulated per hydrogel, were incubated in the Triton X‐100‐based lysis buffer provided in the kit for 1 h at 37°C. The assay was performed following the manufacturer's instructions. Briefly, 50 µL of collected medium was incubated with 50 µL reaction mixture for 30 min at RT in the dark. Afterwards, the reaction was stopped by adding 50 µL stop solution, and the absorbance was measured at 490 nm and 690 nm using a spectrophotometer (CLARIOStar Plus, BMG Labtech).

#### 4.2.13 Biochemical Analysis

To quantify the glycosaminoglycans (GAGs) and DNA within the cell‐hydrogel constructs, samples were collected after 1, 7, 14, and 28 d of culture (*n* = 3 biological donors and *n* = 3 technical replicates per donor per time‐point). The samples were weighed, freeze‐dried, and weighed again to determine their dry weight and water content. Then, gels were digested in hyaluronidase solution (1 mg mL^−1^, Type IV‐S, 3000 U mg^−1^) at 37°C, followed by proteinase K (0.5 mg mL^−1^) digestion at 60°C overnight. The amount of GAGs was measured by a dimethylmethylene blue (DMMB) assay at 525 nm and 595 nm with a spectrophotometer (CLARIOStar Plus, BMG Labtech), using known concentrations of chondroitin sulphate as a reference. DNA content of the samples was measured using a Quant‐iT PicoGreen dsDNA assay kit, according to the manufacturer's instructions.

#### 4.2.14 Cryosectioning

At varying points during culturing, spheroid‐encapsulating hydrogels were washed with PBS for 10 min, before fixation in 4% paraformaldehyde (PFA) for 1 h at RT. Fixated gels were incubated for 20 h in 1 mL 50% Tissue‐Tek O.C.T. compound (Sakura) in PBS at 37°C on a roller bench, before snap‐freezing in liquid nitrogen for 15 min prior to storage at ‐80°C. Hydrogels were then serially sectioned on adherent microscope slides (VWR) at a thickness of 5 µm, allowed to dry at RT overnight and kept at ‐80°C until the samples were used for staining.

#### 4.2.15 Histological Analysis

The proteoglycans secreted by the ACPC spheroids during chondrogenic differentiation in the hydrogel samples were visualized with Safranin‐O staining. The slides were thawed for 20 to 30 min at RT, fixed in methanol (100% v/v, 15 min at RT), and washed with deionized water. Next, they were stained with Weigert's hematoxylin for 10 min, then placed under tap water for 10 min and rinsed in deionized water. They were incubated with 0.4% w/v fast‐green solution for 4 min and rinsed with 1% w/v acetic acid. Upon washing, the samples were incubated with 0.125% w/v safranin‐O solution for 15 min, then washed with 70% ethanol (25 s), and subsequently dehydrated with 96% ethanol (2×), 100% ethanol (2×), xylene, and mounted with Eukitt mounting medium (Sigma‐Aldrich). To assess the deposition of collagen fibers, picrosirius red staining was used. The samples were stained with Weigert's hematoxylin for 10 min, rinsed in tap water, and finally stained with the picrosirius red solution (0.1% w/v Sirius red in saturated aqueous picric acid, Klinipath). The sections were rinsed in 0.01 m HCl, dehydrated with a series of alcohols, and mounted. Microscopic evaluation was performed using an Olympus BX51 microscope.

#### 4.2.16 Immunohistochemistry

The frozen sections were thawed to RT for 30 min, fixed in 100% methanol, and air dried again for 15 min. Next, the sections were washed in PBS and the endogenous peroxidase activity was blocked with 0.3% H_2_O_2_ in PBS for 10 min at RT. After washing 3× in PBS‐Tween20 (0.1%), antigen retrieval was performed using 1 mg mL^−1^ pronase and 10 mg mL^−1^ hyaluronidase for 30 min at 37°C for collagen type I and II staining, while for collagen type X staining the slides were incubated in 1 mg mL^−1^ pepsin in 0.5 m acetic acid for 2 h and in 10 mg mL^−1^ hyaluronidase for 30 min at 37°C. After washing, the samples were blocked in 5% BSA in PBS for 30 min at RT and then incubated with the primary antibodies (collagen I: rabbit monoclonal, 1:100, collagen II: mouse monoclonal, 1:100, and collagen X53: mouse monoclonal, 1:30) diluted in 5% BSA in PBS overnight at 4°C. Hereafter, sections were washed with PBS‐Tween 20 (3 × 10 min) and then incubated with the matching secondary antibody (1:100, Anti‐mouse IgG HRP, and BrightVision+ Anti‐rabbit IgG) for 1 h at RT. Following washing with PBS‐Tween20 (3 × 5 min), the staining (brown) was visualized by DAB oxidation. Finally, the samples were counterstained with Mayer's hematoxylin, washed with water for 10 min, dehydrated, and mounted with Eukitt mounting medium. Images were taken with an Olympus BX51 microscope.

#### 4.2.17 Immunofluorescence Staining

Following methanol fixation, as previously described in Section 4.2.16., the sections were permeabilized with PBS‐Tween20 (0.1%, 3 × 10 min). Next, antigen retrieval was performed by sequential incubation in pronase solution (1 mg mL^−1^) and hyaluronidase solution (10 mg mL^−1^) for 15 min each at 37°C. After each enzymatic treatment, sections were washed 3 × 5 min with PBS‐Tween20 (0.1%). Then, the samples were blocked in 5% BSA in PBS for 30 min at RT, followed by overnight incubation with the primary antibodies (collagen type I: rabbit monoclonal, 1:400; collagen type II: mouse monoclonal, 1:50) diluted in 5% BSA at 4°C. Next, the samples were washed with PBS‐Tween 20 (3 × 10 min), followed by incubation with the matching secondary antibody (Goat anti‐mouse IgG secondary antibody, Alexa Fluor 488, 1:200; donkey anti‐rabbit IgG secondary antibody, Alexa Fluor 594, 1:200) diluted in 5% BSA for 1 h at RT. After washing, the samples were counterstained with DAPI (1:1000 in PBS) to label nuclei, for 10 min at RT, in the dark. Finally, the slides were washed in PBS, mounted with Immu‐Mount and sealed with nail polish.

Imaging of stained samples was conducted on a Thunder microscope (Leica, Germany) using a 10× magnification objective. Imaging parameters for each channel were set based on samples showing high collagen type II or type I fluorescent intensity and were kept consistent across all samples for each channel. At least three replicates were used for each group. Negative controls were used for each slide to ensure absence of autofluorescence. ImageJ2 (Fiji, version 2.14.0/1.55f) was used to analyze the fluorescent images. For each image, channels corresponding to collagen type I and collagen type II were processed separately and thresholded (Otsu method) to segment the positive signal. The thresholded (white) pixels in the binary masks represented the collagen‐positive signal. The number of positive pixels was extracted from the histogram measurements. The collagen type II/collagen type I ratio was then calculated from these values to compare relative protein levels between low‐ and high‐spheroid‐density DA hydrogels.

#### 4.2.18 MEW‐Scaffolds

Polycaprolactone (PCL) MEW scaffolds with a designed box structure that holds inter‐fiber distances of 300 µm and a height of 1 mm (100 layers) were fabricated using a 3D Discovery Evolution (RegenHU, Switzerland). PCL was heated to 80°C and extruded through a grounded 25G nozzle at a pressure of 0.1 MPa, using a voltage range of 5.0‐5.5 kV, a collector distance of 2.8 mm, and a collector translational speed of 16 mm s^−1^. G‐code was used to control the printing parameters as well as the motion of the printhead and the printed pattern. After printing, a biopsy punch (diameter = 6 mm) was used to collect samples from the scaffold for further experiments. To increase hydrophilicity, the scaffolds used for cell culture were treated with 1 m NaOH for 30 min, washed five times in PBS, and subsequently in 70% ethanol, then left to evaporate. For gel casting, custom‐made cylindrical PDMS molds (6 mm × 1 mm) were created. The scaffolds were placed in the molds and cast with gel directly on top of the scaffold. The mold was clamped down with a glass slide and incubated at 37°C for 4 h.

#### 4.2.19 Statistical Analysis

All statistical analyses were conducted using Graphpad Prism 10.4 software. Data is reported as mean ± standard deviation, and significance for all performed analyses was determined at *p* < 0.05 (*). Comparisons between two groups were made via unpaired t‐tests with assumed Gaussian distribution. For comparisons with more than two groups, one‐way ANOVAs were carried out with a Tukey's honest significant difference (HSD) post hoc test. Ordinary two‐way ANOVAs were performed with α < 0.05 and a Tukey's HSD post hoc test for multiple comparisons.

## Funding

Nederlandse Organisatie voor Wetenschappelijk Onderzoek, NWO/VICI 18673 and NWA.1389.20.192.

## Conflicts of Interest

The authors declare no conflicts of interest.

## Supporting information




**Supporting File**: adhm70956‐sup‐0001‐SuppMat.docx.

## Data Availability

The data that support the findings of this study are available on https://doi.org/10.24416/UU01‐3ZUIML
