# Supplementary material for: Diels‐Alder Click Chemistry as a Dynamic‐Covalent Crosslinking Method in Spheroid‐Encapsulating Hydrogels for Cartilage Engineering
Source: Adv Healthc Mater. 2026 Feb 20;15(17):e05013. doi: 10.1002/adhm.202505013 (PMC13175297; doi:10.1002/adhm.202505013)
Supplement: Supplementary file 1 — Supporting File: adhm70956‐sup‐0001‐SuppMat.docx. [file ADHM-15-0-s001.docx]

Supporting Information

**Diels-Alder click chemistry as a dynamic-covalent crosslinking method in spheroid-encapsulating hydrogels for cartilage engineering**

Sanne M. van de Looij^1^, Antonia G. Vasilopoulou^2,3^, Lennard Spauwen^2,3,4^, Antoinette van den Dikkenberg^1^, Jasmijn V. Korpershoek^2,5^, Mylene de Ruijter^2,3,6^, Jos Malda^2,3,6^, Bas G.P. van Ravensteijn^1^, Tina Vermonden^1,3*^

^1^ Division of Pharmaceutics, Utrecht Institute for Pharmaceutical Sciences (UIPS), Utrecht University, Universiteitsweg 99, 3584 CG Utrecht, The Netherlands

^2^ Department of Orthopedics, University Medical Center Utrecht, Heidelberglaan 100, 3584 CX Utrecht, The Netherlands

^3^ Regenerative Medicine Center Utrecht, Uppsalalaan 8, 3584 CT Utrecht, The Netherlands

^4^ HU University of Applied Sciences Utrecht, Padualaan 99, 3584 CH Utrecht, The Netherlands

^5^ Department of Orthopedic surgery, Mayo Clinic Rochester, Minnesota, USA

^6^ Department of Clinical Sciences, Faculty of Veterinary Medicine, Utrecht University, Yalelaan 1, 3584 CL Utrecht, The Netherlands

* Corresponding author: t.vermonden@uu.nl


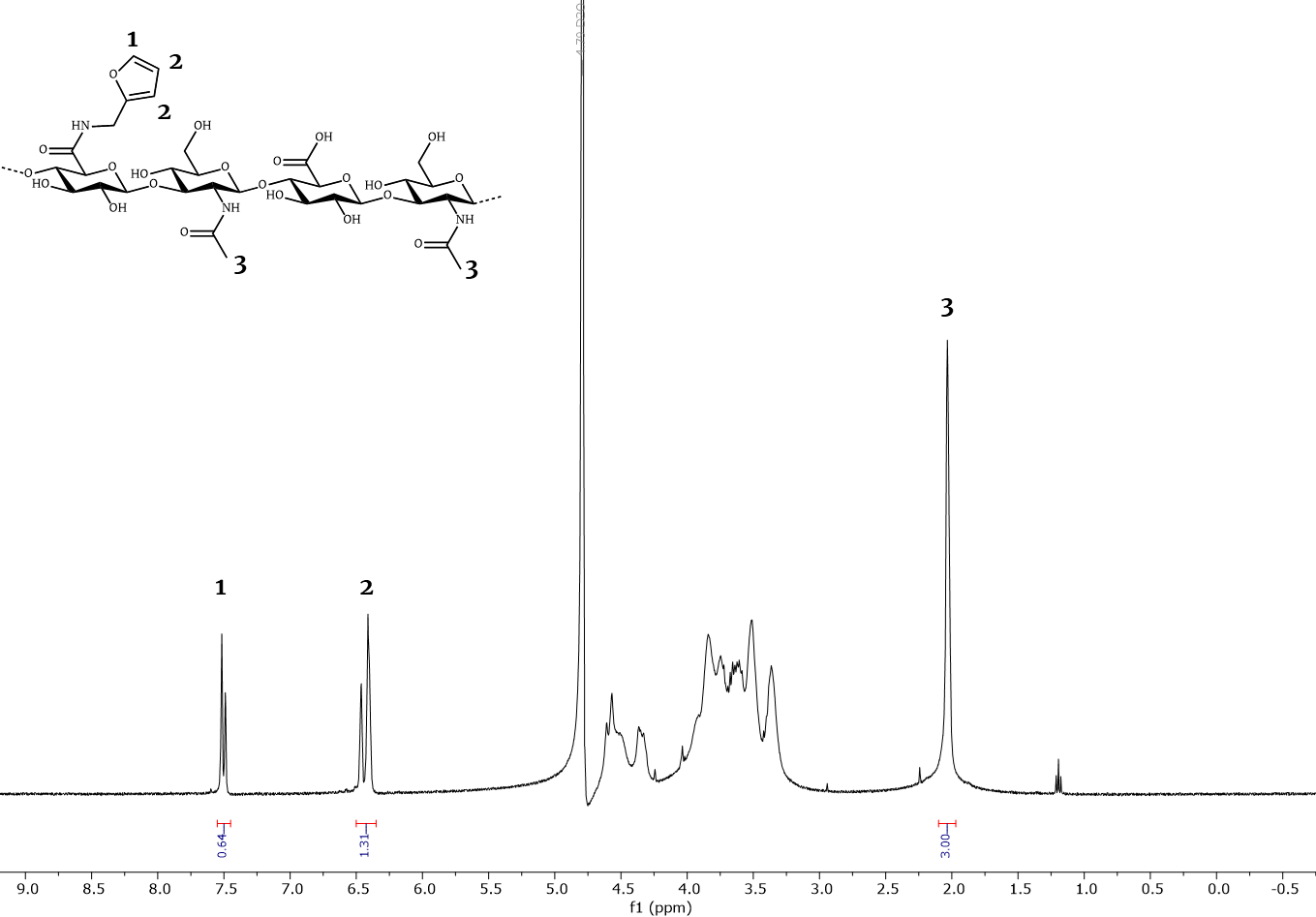


***Figure S1****: ^1^H-NMR spectrum of HA modified with furan groups (HAFU, 381 kDa). Signals at 7.5 ppm and 6.4 ppm correspond to aromatic furan signals, the signal at 2.1 ppm refers to the N-acetyl glucosamine unit on the HA backbone. Degree of functionalisation was determined as* ${(\int7.5 ppm+ \int6.4 ppm)}/{\int2.1 ppm}*100\%$


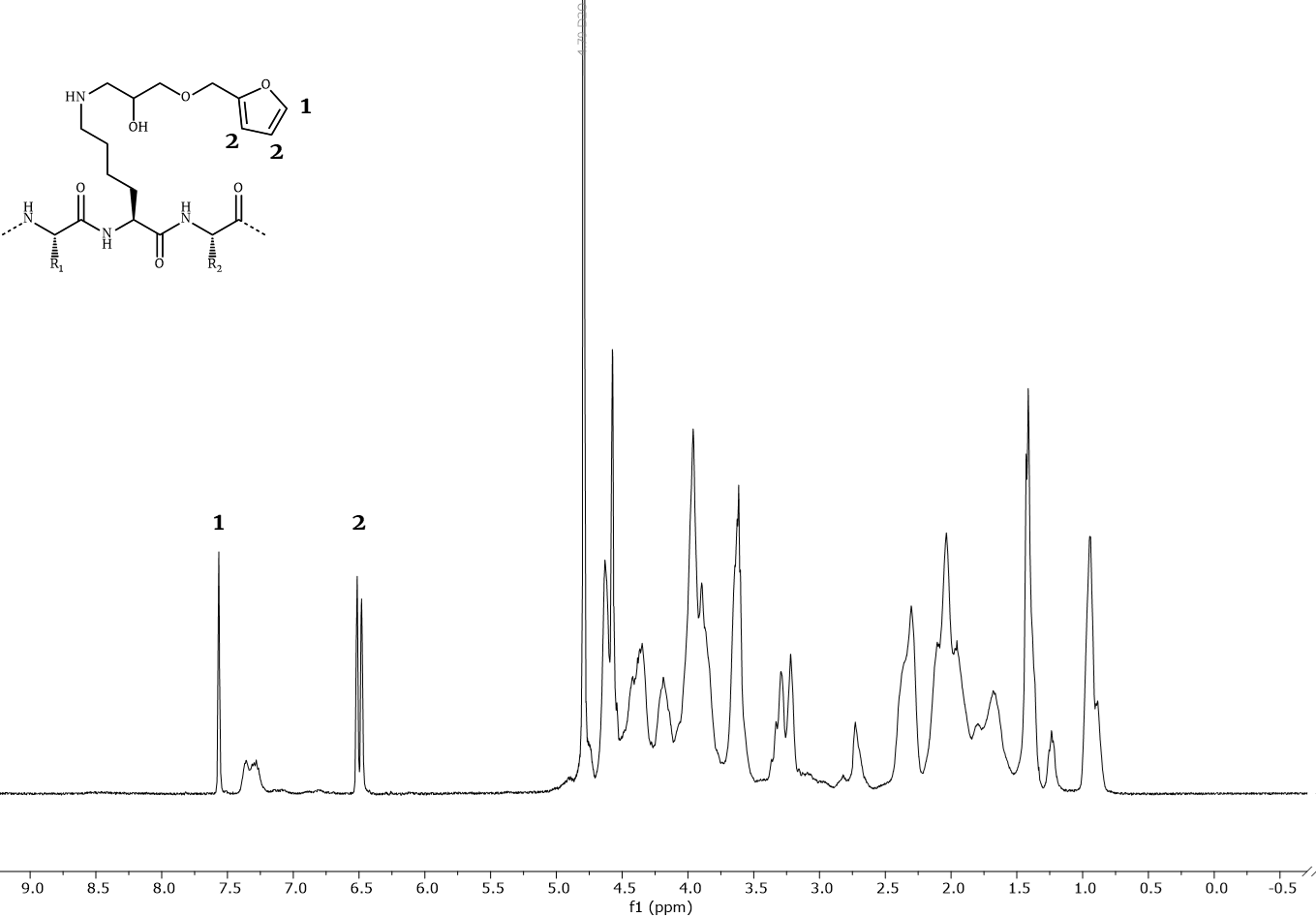


***Figure S2:*** *^1^H-NMR spectrum of Gelatin modified with furan groups (GelFU). Peaks at 7.6 ppm and 6.5 ppm correspond to aromatic furan signals.*

***Figure S3****: Representive oscillation amplitude experiment showing a linear viscoelastic region up to ~ 70% strain. Values represent the average ± st.dev of n = 2 replicates, formulation 5.5% TPC in PBS.*

***Figure S4****: Effect of cystine (0.2 mM) on gelation time and storage modulus of 5.5% TPC in PBS. Bars represent mean ± stdev of n = 3 technical replicates.*


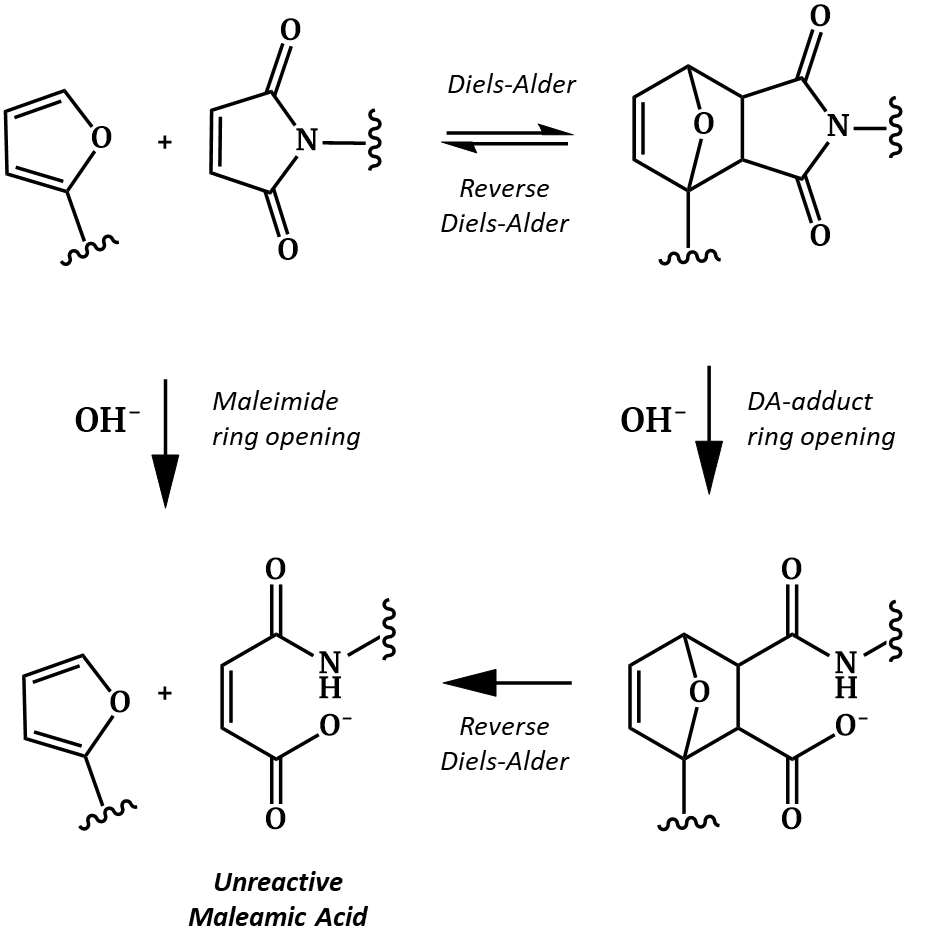


***Figure S5****: Graphical depiction of maleimide degradation in Diels-Alder reactions.*

***Figure S6****: Cell viability measured by metabolic activity of adherent equine ACP cells one day after 4 h incubation at various pH values. Error bars represent st.dev of n = 5 technical replicates.*


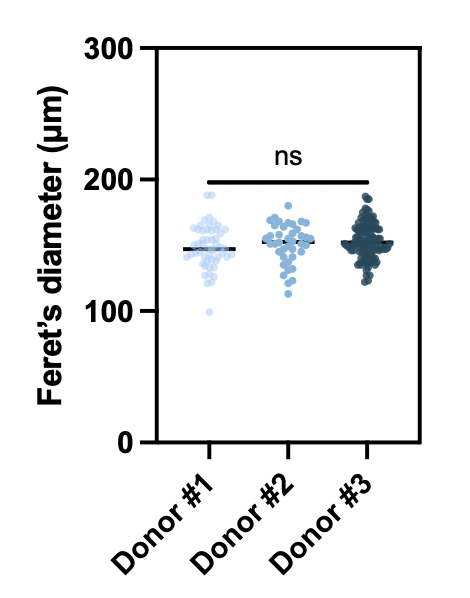


***Figure S7:*** *Comparison of average spheroid diameter (Feret’s diameter) on day 3, prior to hydrogel encapsulation, for the three donors used in this study. No statistically significant differences were detected among donors.*


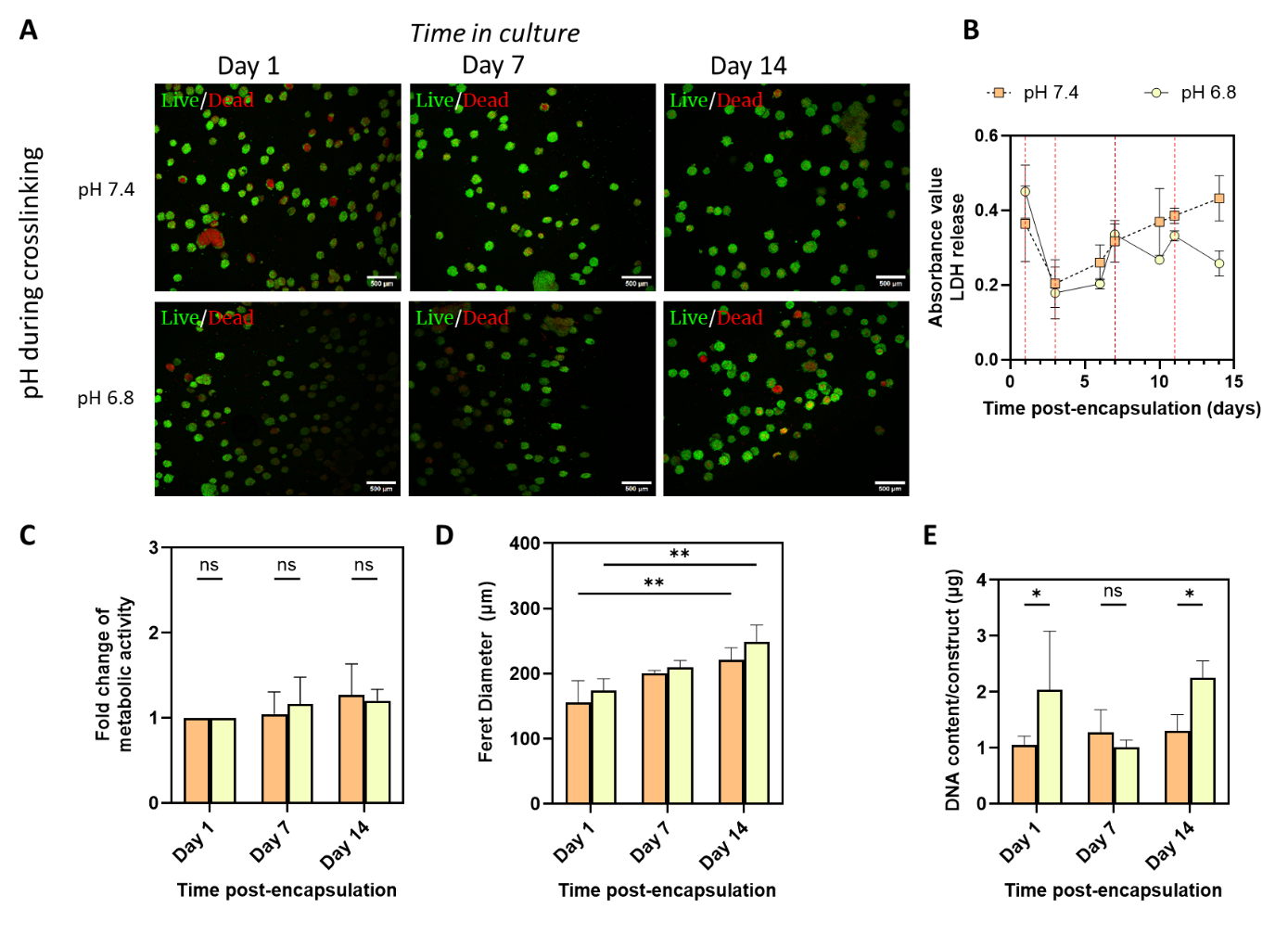


***Figure S8****: Encapsulation of ACPC spheroids shows good biocompatibility of HA/Gelatin/PEG-based DA hydrogels crosslinked in pH-altered medium at a low spheroid concentration. (A) Confocal microscopy images of encapsulated ACPC spheroids with calcein AM (live)/propidium iodide (dead) staining at various timepoints after crosslinking at either pH 7.4 or pH 6.8. All samples were treated equally after crosslinking. Scale bars represent 500 µm. (B) LDH release, (C) Metabolic activity, (D) Feret diameter and (E) DNA content of encapsulated ACPC spheroids over time after crosslinking. In all graphs, values represent the average ± st.dev. of n = 3 technical replicates, donor #2. Statistical significance in bar graphs is denoted as * (p < 0.05), ** (p < 0.01), *** (p < 0.001) or **** (p < 0.0001).*


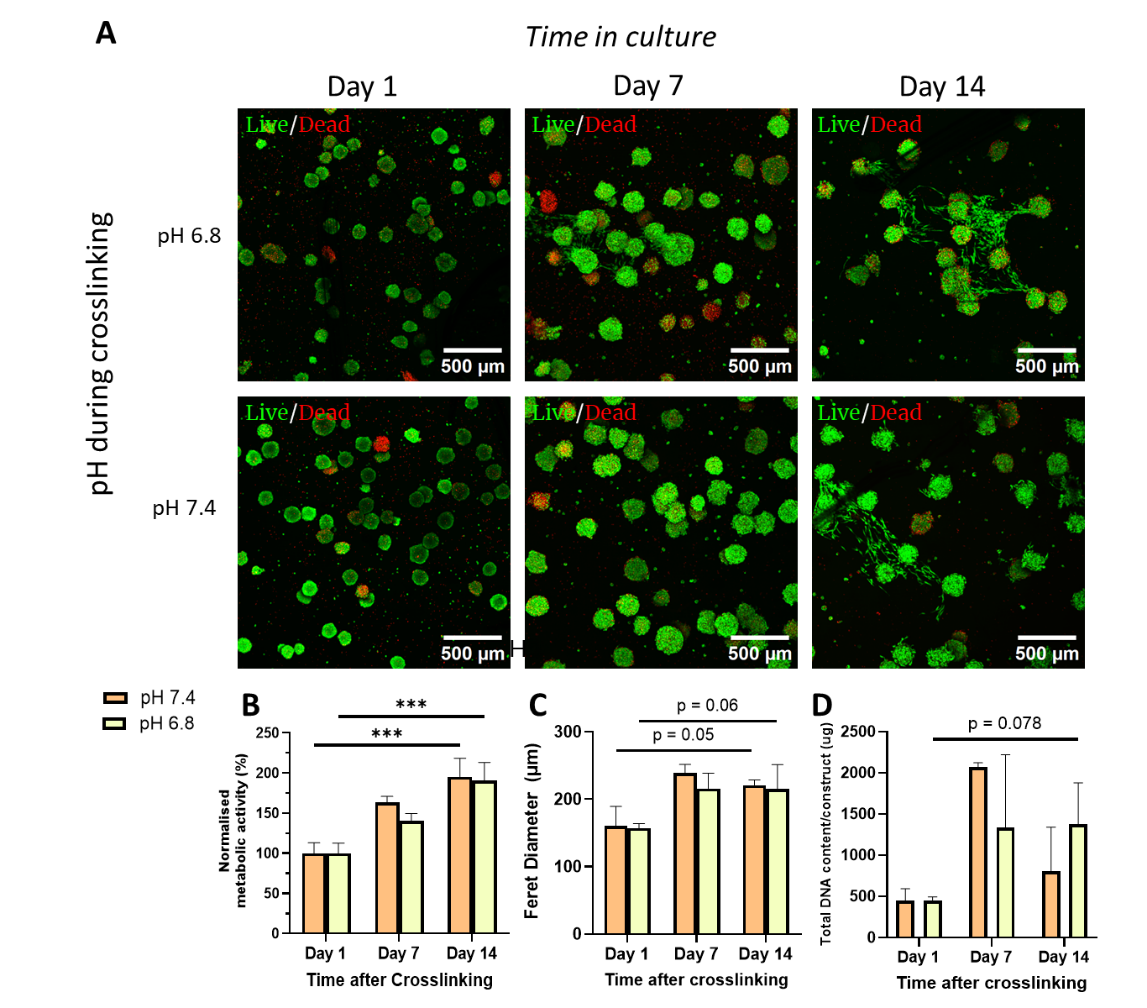


***Figure S9:*** *Encapsulation of ACPC spheroids shows good biocompatibility of HA/Gelatin/PEG-based DA hydrogels crosslinked in pH-altered medium at a low spheroid concentration. (A) Confocal microscopy images of encapsulated ACPC spheroids with calcein AM (live)/propidium iodide (dead) staining at various timepoints after crosslinking at either pH 7.4 or pH 6.8. All samples were treated equally after crosslinking. Scale bars represent 500 µm. (B) Metabolic activity, (C) Feret diameter and (D) DNA content of encapsulated ACPC spheroids over time after crosslinking. In all graphs, values represent the average ± st.dev. of n = 3 technical replicates, donor #1. Statistical significance in bar graphs is denoted as * (p < 0.05), ** (p < 0.01), *** (p < 0.001) or **** (p < 0.0001).*


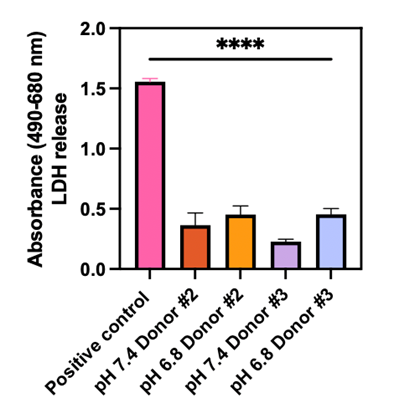


***Figure S10:*** *LDH-release on day 1 post-encapsulation at a low spheroid concentration indicates minimal cell damage in DA hydrogels crosslinked at pH 7.4 and pH 6.8. LDH was measured for two donors on day 1 and compared to a positive control, where complete lysis of the same number of encapsulated spheroids on day 0 resulted in maximum LDH release. Data presented as average ± st.dev. of n = 4 technical replicates for the positive control and n = 3 technical replicates per donor.*


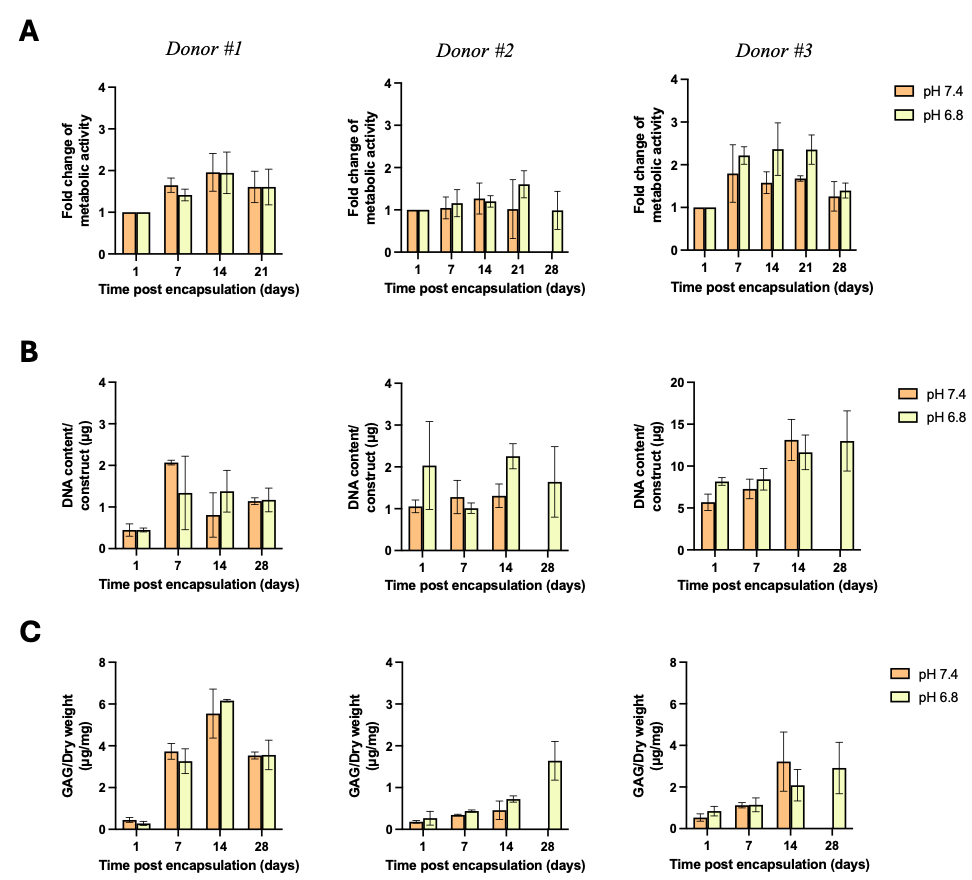
***Figure S11:*** *Encapsulation of ACPC spheroids in DA hydrogels at a low spheroid concentration crosslinked at different pH conditions results in comparable biological activity. (A) Metabolic activity, (B) DNA content, and (C) GAG content normalized over the dry weight of the samples, were quantified for all three donors over a 28-day culture period. In all graphs, values represent the average ± st.dev. of n = 3 technical replicates per donor.*

***Table S1:*** *Measurements of absorbance values following DMMB assay for GAG quantification in cell-free DA hydrogels, crosslinked at pH 6.8. Absorbance values of blank controls were substracted from all measurements.*

| **Time post-encapsulation** | **Absorbance values (525/595 nm)** | | |
| --- | --- | --- | --- |
| Day 1 | -0.0067891 | -0.0123888 | -0.0144782 |
| Day 7 | -0.0024285 | -0.0059509 | -0.0086108 |
| Day 14 | -0.0082241 | -0.0038353 | -0.0029078 |
| Day 28 | -0.0070044 | -0.7346574 | -0.740332 |


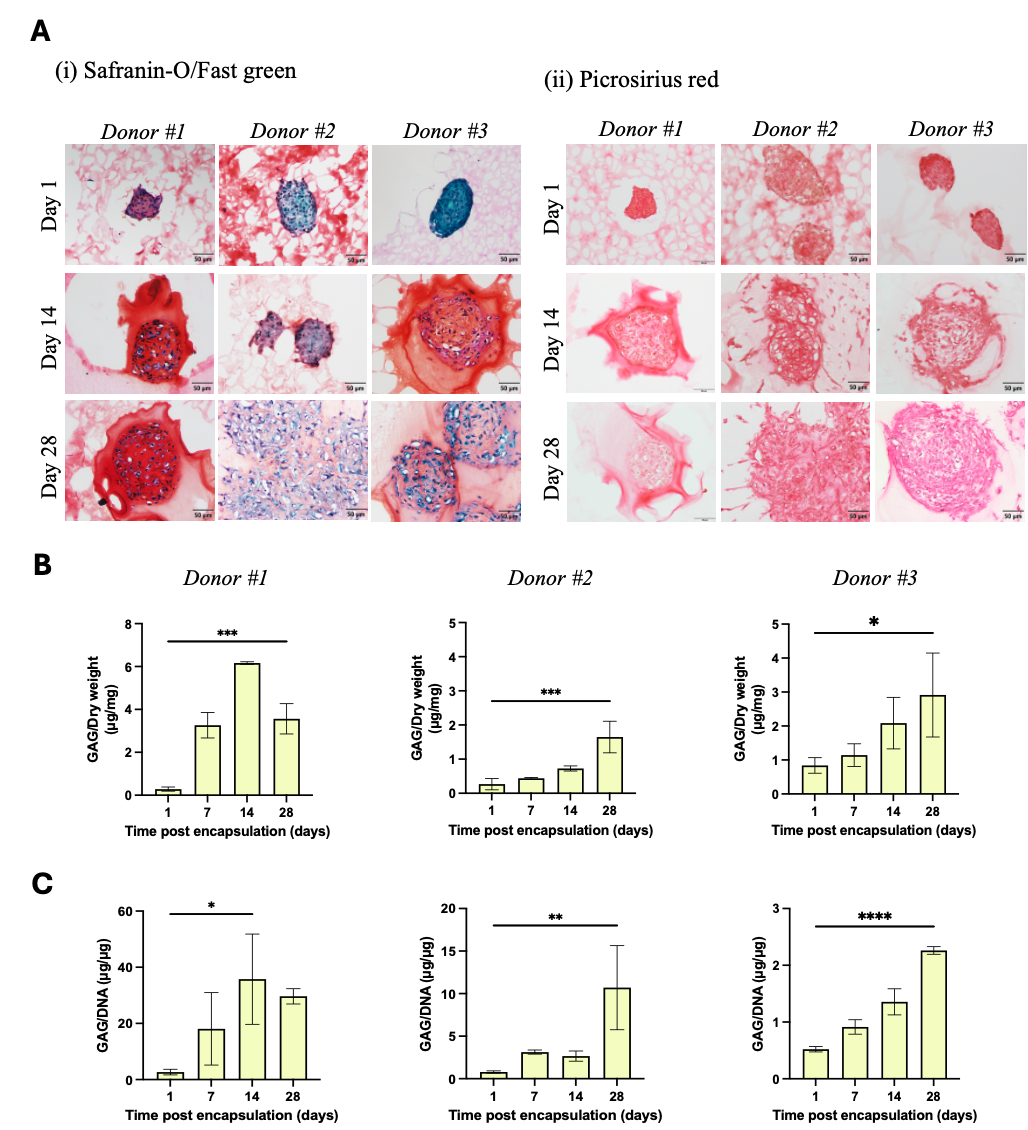


***Figure S12:*** *Histological evaluation and biochemical analysis of ECM deposition by equine ACPC spheroids encapsulated at a low spheroid concentration in DA hydrogel crosslinked at pH 6.8 over a 28-day culture period. The DA hydrogel supports chondrogenic differentiation across all three donors tested, as shown by (Ai) positive Safranin-O and (Aii) Picrosirius red staining, along with increased GAG deposition, quantified using the DMMB assay and normalized over (B) dry weight or (C) DNA content. In all graphs, values represent the average ± st.dev. of n = 3 technical replicates per donor.*


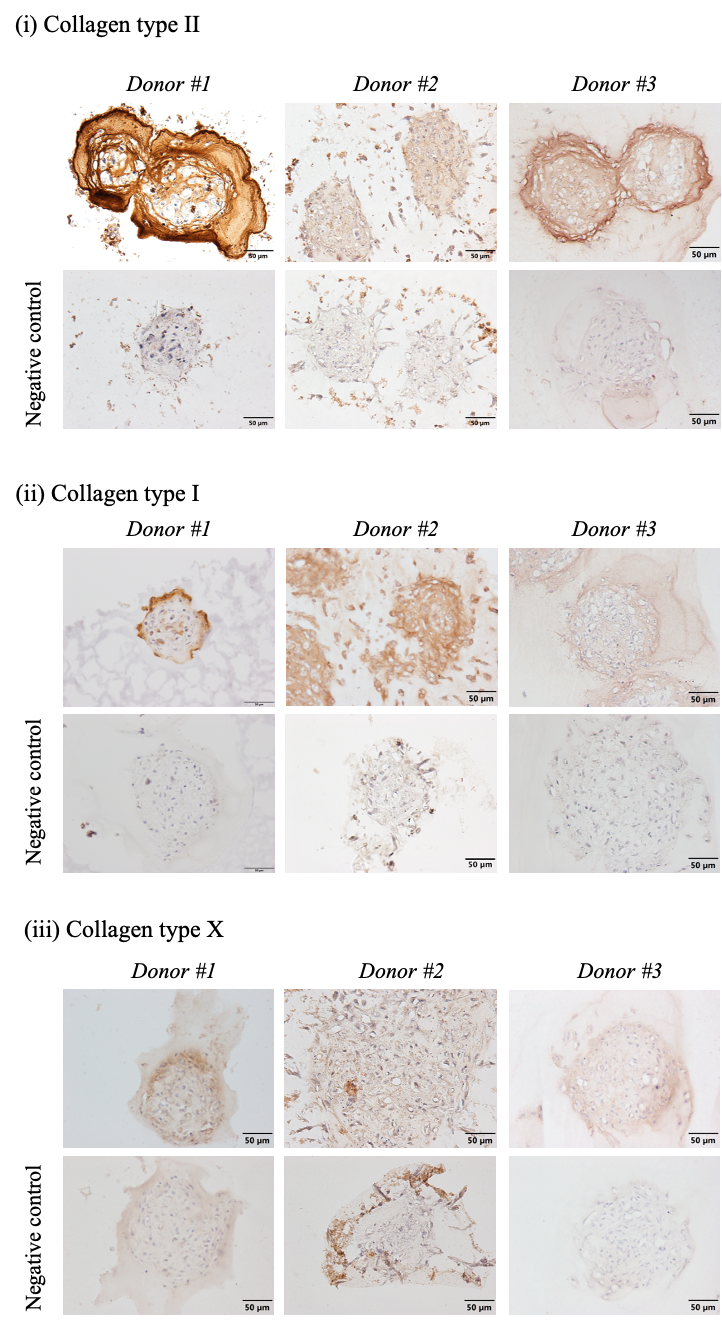


***Figure S13:*** *Immunohistochemical staining for collagen types II, I, and X of equine ACPC spheroids encapsulated at a low concentration in DA hydrogels crosslinked at pH 6.8 on day 28. All donors stained positive for both collagen types II and I, and weakly stained for collagen type X.*


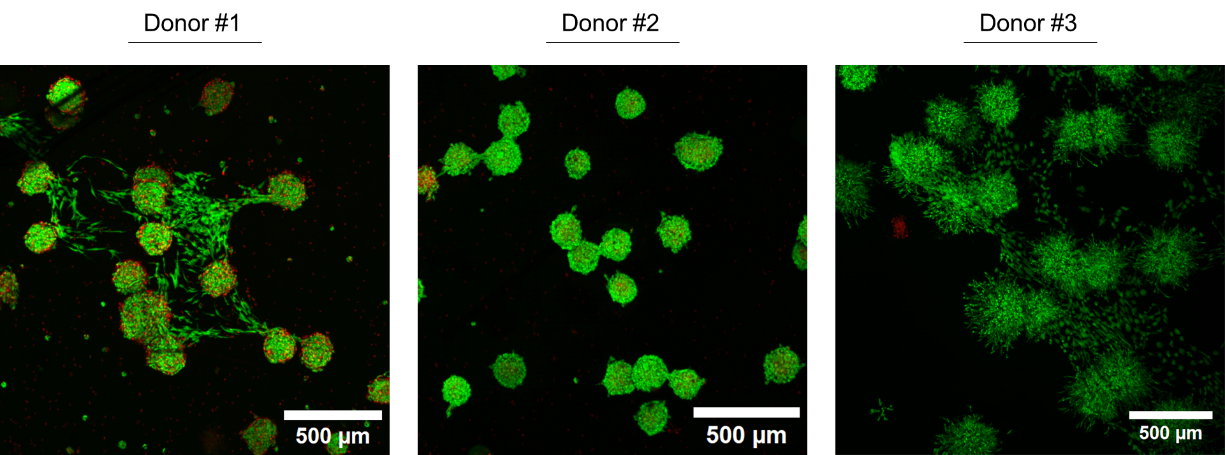


***Figure S14:*** *Live/dead staining of encapsulated ACPC spheroids at low spheroid concentration, showing a migratory pattern without clear fusion on day 14 after encapsulation. Scale bars represent 500 µm.*


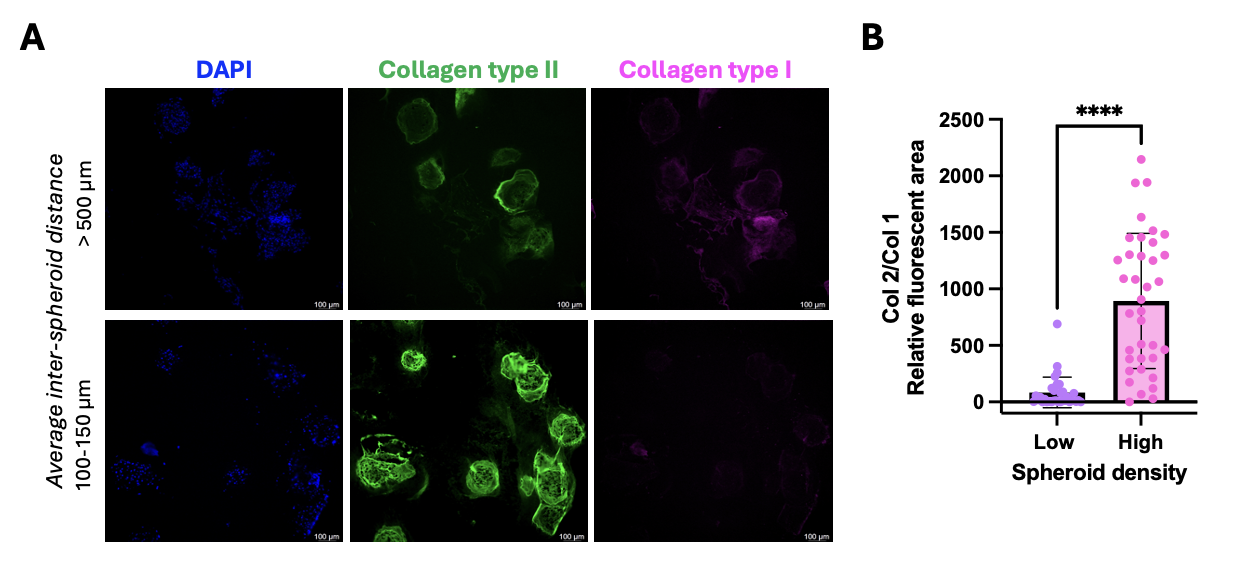


***Figure S15:*** *(A) Immunofluorescent staining of DAPI (blue), collagen type II (green), and collagen type I (magenta) in equine ACPC spheroids encapsulated in DA hydrogel crosslinked at pH 6.8 in low and high spheroid densities. (B) Quantification of the relative fluorescent area ratio of collagen type II to collagen type I.*

***Figure S16:*** *Construct swelling after 24 hours in medium in DA hydrogels (left) and DA hydrogels reinforced with a MEW scaffold (right). Statistical significance in bar graphs is denoted as **** (p < 0.0001).*


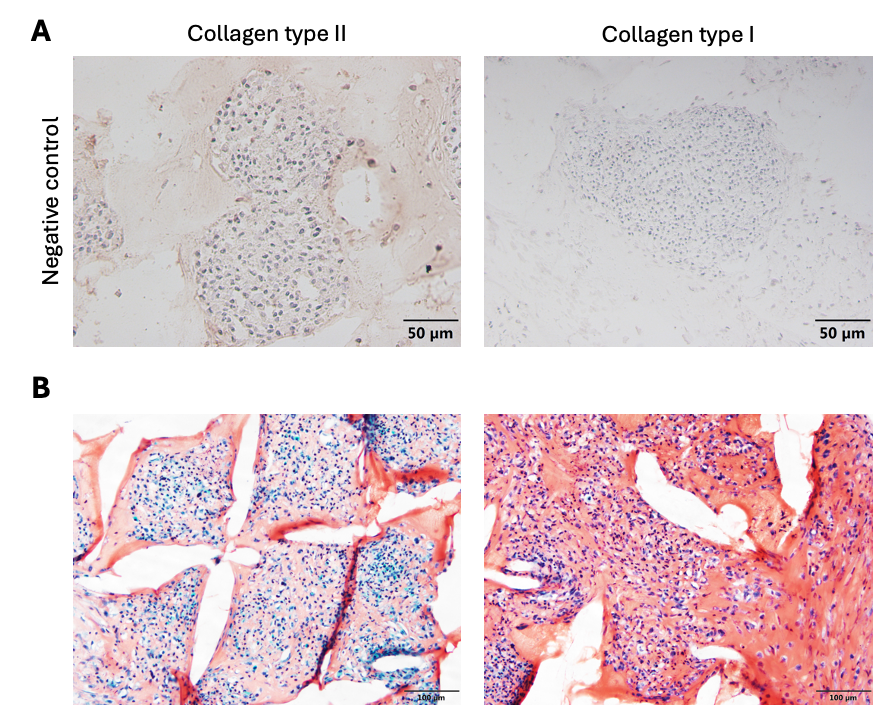


***Figure S17:*** *(A) Negative controls for immunohistochemical staining for collagen types II and I of ACPC spheroids encapsulated at a high spheroid concentration in MEW-reinforced DA hydrogels on day 28. (B) Safranin-O/Fast green staining shows that the ACPC spheroids filled the MEW mesh boxes and largely fused, depositing a matrix rich in GAGs.*


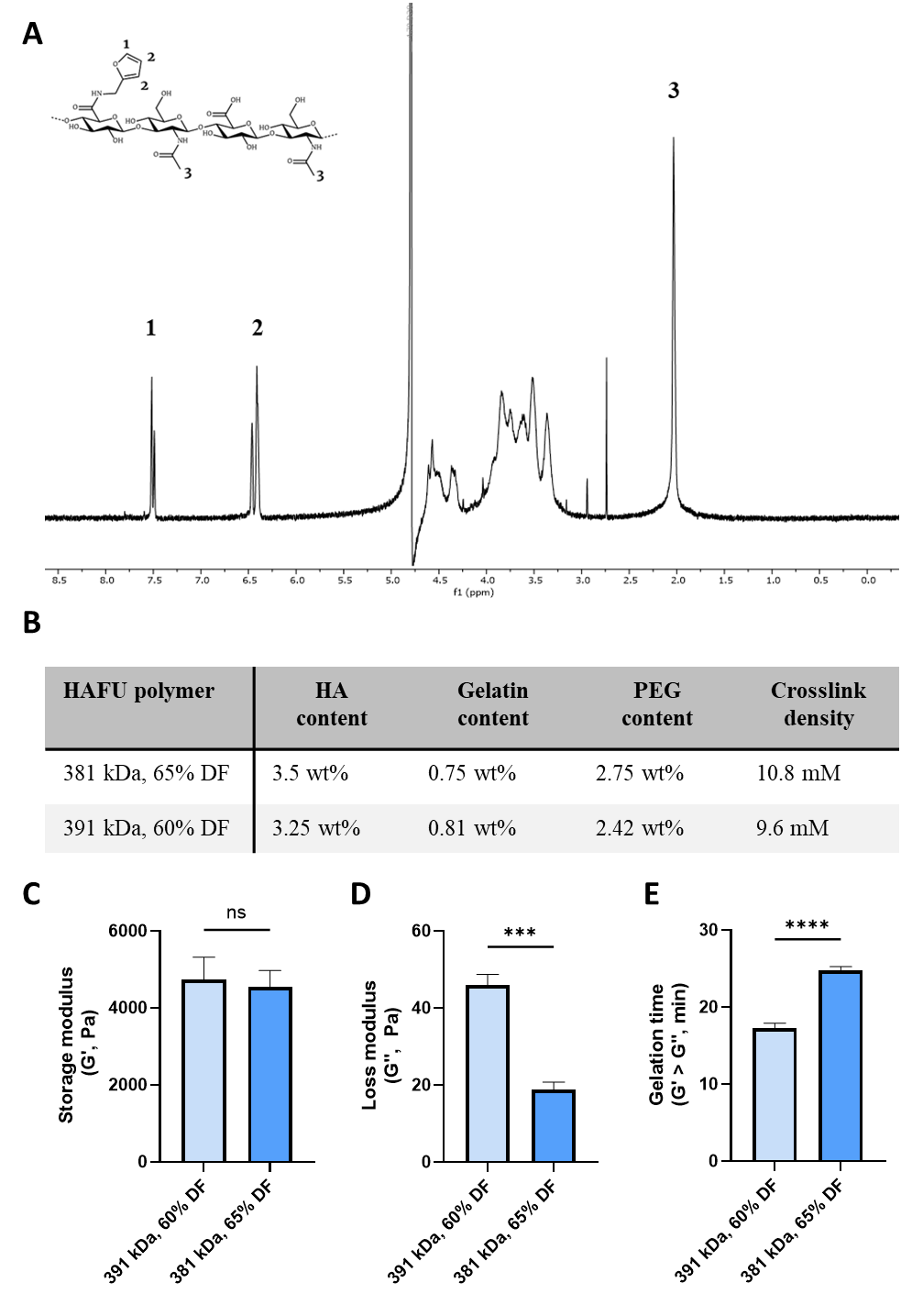


***Figure S18:*** *(A) ^1^H-NMR spectrum of HA modified with furan groups (HAFU, 391 kDa). Signals at 7.5 ppm and 6.4 ppm correspond to aromatic furan signals, the signal at 2.0 ppm refers to the N-acetyl glucosamine unit on the HA backbone. Degree of functionalisation was determined as* ${(\int7.5 ppm+ \int6.4 ppm)}/{\int2.0 ppm}*100\%$*. (B) Comparison of hydrogel formulations, (C) Storage Modulus (G’), (D) Loss modulus, and (E) gelation time using HAFU 381 kDa, 65% DF and HAFU 391 kDa, 60% DF. In all graphs, values represent the average ± st.dev. of n = 3 technical replicates. Statistical significance in bar graphs is denoted as *** (p < 0.001) or **** (p < 0.0001). Hydrogel formulations were considered equal in storage modulus, in the same order of magnitude in loss modulus and slightly different in gelation time.*
